# Epistasis Is a Major Determinant of the Additive Genetic Variance in *Mimulus guttatus*

**DOI:** 10.1371/journal.pgen.1005201

**Published:** 2015-05-06

**Authors:** Patrick J. Monnahan, John K. Kelly

**Affiliations:** Department of Ecology and Evolutionary Biology, University of Kansas, Lawrence, Lawrence, Kansas, United States of America; Swedish University of Agricultural Sciences, SWEDEN

## Abstract

The influence of genetic interactions (epistasis) on the genetic variance of quantitative traits is a major unresolved problem relevant to medical, agricultural, and evolutionary genetics. The additive genetic component is typically a high proportion of the total genetic variance in quantitative traits, despite that underlying genes must interact to determine phenotype. This study estimates direct and interaction effects for 11 pairs of Quantitative Trait Loci (QTLs) affecting floral traits within a single population of *Mimulus guttatus*. With estimates of all 9 genotypes for each QTL pair, we are able to map from QTL effects to variance components as a function of population allele frequencies, and thus predict changes in variance components as allele frequencies change. This mapping requires an analytical framework that properly accounts for bias introduced by estimation errors. We find that even with abundant interactions between QTLs, most of the genetic variance is likely to be additive. However, the strong dependency of allelic average effects on genetic background implies that epistasis is a major determinant of the additive genetic variance, and thus, the population’s ability to respond to selection.

## Author Summary

Complex traits are influenced not only by the effects of individual genes but also by the myriad ways that these genes interact with one another, commonly referred to as epistasis. Theory suggests that epistasis could have important population-level implications in terms of the genetic variance components that govern evolution in response to natural or artificial selection. Unfortunately, empirical examples extending from observed interactions between genes to genetic variances are scant, particularly for natural populations. Here, we characterize epistasis between naturally segregating polymorphisms in *M*. *guttatus* and determine the cumulative effect of epistasis on population genetic variance components. To do this, we first elaborate the necessary statistical theory to accommodate estimation error in genetic effects, as failing to do so will upwardly bias variance predictions. We find that gene interactions have a net positive effect on both the total and additive genetic variance for most traits; however, the contribution of individual loci to the additive variance depends heavily on the genotype frequencies at other loci. Therefore, the effect of epistasis extends beyond the individual’s phenotype to influence how both populations and their component alleles respond to selection.

## Introduction

Epistasis, the interactions between genetic loci, is an important determinant of phenotypes across a large number of taxa [1–8]. Yet for quantitative (complex) traits, the net effect of epistasis on the components of variation, specifically the additive genetic variance (*V*_*A*_) that determines the response to natural or artificial selection, remains polemical. This is evidenced by a renewed debate over the evolutionary relevance of epistasis as exemplified by Crow [9] and Hansen [10]. An unfortunate source of confusion sustaining this debate is the simultaneous use of terms to describe both the effects of individual genes as well as the genetic variance components of populations (additive, dominance, and epistatic). It has long been known that high additive genetic variance does not imply additive gene action [11], a conclusion reiterated by the theoretical demonstration in Hill *et al*. [12] (see also [13]). However, there is little empirical evidence regarding the extent to which gene interactions determine the additive genetic variance, [14–17], which leaves several important questions unanswered. For instance, do interactions among genes tend to increase or decrease *V*_*A*_ of traits, on average? As allele frequencies change in response to selection, does epistasis accelerate or dampen the corresponding change in *V*_*A*_?

For a particular locus, the contribution to *V*_*A*_ depends on the average effect of substitution at the locus and on the frequencies of the different alleles in the population [11]. The total *V*_*A*_ is a simple sum over all loci affecting the trait. With epistasis, the average effect of a locus will change as the frequency of its epistatic partners in the population change [16]. Thus, the contribution of a locus to *V*_*A*_ depends simultaneously on its own allele frequencies as well as the allele frequencies of all other segregating loci. Association mapping studies can estimate the *V*_*A*_ contributed by a locus (e.g [7]), but this estimate is an average over the genetic backgrounds in the population. The extent to which locus-specific *V*_*A*_ is determined by interactions with other loci remains unknown. An alternative to association mapping is to estimate genetic effects from genotypes produced from experimental crosses, with the remainder of the genetic background held constant. These genetic effects, often termed functional effects [18], can be defined as deviations from a reference genotype, and therefore, do not depend on an unknown distribution of genetic backgrounds. Given allele frequencies in a population, it is straightforward to calculate total and locus-specific variances based on these genetic effects. One can also calculate these variances under the assumption of additivity of loci (i.e. no epistasis). Contrasting these variances to those calculated from genetic effects based on multi-locus measurements provides a simple, direct demonstration of the effect of epistasis on *V*_*A*_ [15]. Further, one can observe how this contrast changes as population allele frequencies change.

Standard equations used to calculate *V*_*A*_ from genetic effects [11] assume that effects are estimated without error. Estimation error in genetic effects is often substantial even with large sample sizes, and failing to account for this error will result in an upward bias in variance predictions [19]. This is because genetic effects are squared and different effects are multiplied together when variances are calculated. As the true value for a genetic effect is the estimate minus a residual (the estimation error), treating the genetic effect estimates as the truth results in the inclusion of squared residual terms thus biasing variance components upwards. Luo *et al*. [19] derived a correction that incorporates the variance-covariance matrix of the genetic effect estimates for the case of a single locus. Here, we extend the bias-correction to multiple loci, accommodating epistatic terms, and demonstrate its validity using parametric bootstrapping. Then, we use the corrected variances to explore the effects of epistasis on the total and additive genetic variances under different models of allele frequency.

We consider loci affecting floral morphology and development rate that are polymorphic within a single population of *Mimulus guttatus* (yellow monkeyflower). *Mimulus guttatus* is an emerging model in evolutionary genetics; notable for its high degree of phenotypic and genetic variation within and between populations as well as its ability to adapt to novel environments [20]. The species is broadly distributed across western North America and ranges from high alpine to low-elevation coastal environments. In *Mimulus guttatus*, multiple studies have shown that epistasis contributes to within-population variation in floral morphology, development time, and fitness components [3, 21]. Interaction effects are routinely of the same magnitude as single-locus effects, although the magnitude and direction of epistasis between loci is highly variable.

In this study, we maximize statistical power to estimate direct and epistatic effects between QTL using Double-NIL lines (DNILs, hereafter), in which two loci segregate in an otherwise isogenic genetic background. We confirm the finding of important but highly variable epistasis between these QTL, but also quantify the contribution of epistasis to population genetic variance using the genetic effect estimates and a model of allele frequency. We find that the average effect, which determines the locus-specific response to selection, depends heavily on genetic background or rather the frequency of different genetic backgrounds in the population (i.e. the genotypes at all other loci). For some traits, this leads to an average increase in genetic variance components, whereas in others, the effect is opposite albeit minimally. Overall, it is clear that epistasis is an important determinant of both individual phenotype as well as the genetic variance components, which govern the ability of a population to respond to natural or artificial selection.

## Materials and Methods

### Genetic lines

The DNILs were derived from a previous study by Kelly and Mojica [3]. The process of mapping the original QTL began with a large-scale artificial selection experiment on a collection of lines derived from a single natural population at Iron Mountain in Central Oregon. This resulted in populations with highly divergent floral traits [22]. Individuals from the tails of the distribution were randomly selected and crossed to produce three F1 populations and each of these populations were backcrossed for six generations to IM767, a commonly used inbred line with medium floral trait values derived from the same natural population [23]. This resulted in 3 panels of Nearly Isogenic Lines (NILs; 493 NILs in total), with each NIL containing a random segment of donor genome from one of the three F1 populations. NILs were measured for corolla width, and selective genotyping of NILs from the tails of the distribution identified 7 QTLs affecting corolla width. Three rounds of background-cleaning were performed for each of the 7 NILs, using a combination of selfing and backcrossing to IM767 to eliminate segments of donor, non-IM767 genome from other parts of the genome.

The seven NILs containing the QTL were crossed in each possible pairing to produce 21 F1’s each of which contained solely the double-heterozygote for the donor alleles present in their parents. A single individual from each F1 was self-fertilized, and the resulting F2’s were genotyped at the relevant loci (see S1 Table for the list of diagnostic markers for the 7 QTL). The four true-breeding (double-homozygous) genotypes from each F2 were set aside and self-fertilized, in order to serve as the parents of a single Double-NIL line set. Each DNIL is essentially a collection of nine genotypes corresponding to two biallelic QTL segregating in an otherwise uniform genetic background. For a single DNIL, we create these nine genotypes by selfing the true-breeders as well as crossing them in all possible directions to produce the five heterozygous genotypes. With seven QTL, there are 21 possible pairs of loci; however, this study examines only 11 of the DNILs owing to loss of several lines used in Kelly and Mojica [3].

### Phenotype data

Plants were grown in the University of Kansas greenhouse in five large cohorts. All nine two-locus genotypes for a particular Double-NIL were included in a cohort, which meant that only a subset of Double-NILs could be included in any one cohort. Within a cohort, multiple seed families from each genotype were sprinkled into unique 2x2 in. pots and watered generously. After approximately 10 days, individual plants were transplanted to their own 2x2 in pot. The pot locations were randomized initially and rotated regularly to avoid effects of inconsistent conditions within the greenhouse. Plants were watered every other day following transplant and fertilized once a week.

Upon flowering, plants were measured for several traits: corolla width (CW), distance between stigma and anther (SA separation), pistil length, and the number of days until first flower (DTF). Measurements were taken on all open flowers present at time first flower, which was typically only one or two. The corolla width is the widest distance of the flattened width of the lower lip of the corolla, while the rest of the measurements are self-explanatory. Kelly and Mojica [3] measured the double-homozygotes for 17 of the 21 DNILs. For this study, we elaborated measurements to include all nine two-locus genotypes for 11 of the 21 DNILs. As there is significant overlap between individuals in this and the aforementioned study, we combined the relevant data from Kelly and Mojica [3], which included plants grown in seven distinct cohorts. This resulted in a highly unbalanced dataset, but provided greater accuracy for estimating particular genotypic means. All individuals were grown at the KU greenhouse under the same watering and fertilizer regiment.

### Confirmation genotyping

Crossing two of the true-breeding genotypes within a DNIL will necessarily result in genetically identical heterozygous offspring; however, we genotyped a subset of individuals from each cohort via touch-down PCR at gene-based markers diagnostic of particular QTL (S1 Table) in order to confirm that progeny genotypes were as expected (incorrect genotypes occasionally result from mislabeling or accidental pollen transfer during selfing/crossing). The few individuals with incorrect genotypes that were identified (typically < 5% per cohort) were removed from the analysis along with all of their siblings. PCR fragments were analyzed on ABI 3130 BioAnalyzer, and genotype calls were made using GeneMapper (Applied Biosystems, Foster City, CA, USA).

### The overall test for epistasis

We performed a likelihood ratio test to compare a full and reduced model for each trait corresponding to models with and without epistatic parameters, respectively. In R, we fit each model using REML as implemented in the “lme4” package followed by a call to the “anova.merMod()” function [24]. This produces a likelihood for each model from which a likelihood ratio is calculated and compared to a Chi-squared distribution with degrees of freedom equal to the difference in the number of parameters between the full and reduced model. There were 60 degrees of freedom in the full model and 16 in the reduced model, which corresponds to the number of genetic effects plus the cohort and family effect. The difference, 44, is the degrees of freedom for each of the likelihood ratio tests.

### Linear model for estimation of genetic effects

We estimated the single-locus and epistatic genetic effects using the NOIA *functional* genetic effect model [18]:

$$\begin{array}{l} Z_{ijklmn}=\mu +a_{i}X_{a_{i}}+d_{i}X_{d_{i}}+a_{j}X_{a_{j}}+d_{j}X_{d_{j}}+aa_{ij}X_{a_{i}}X_{a_{j}}\\ +ad_{ij}X_{a_{i}}X_{d_{j}}+da_{ij}X_{d_{i}}X_{a_{j}}+dd_{ij}X_{d_{i}}X_{d_{j}}+C_{k}+F_{l}[G_{m}]+\varepsilon_{ijklmn} \end{array}$$ (1)

where

$$X_{a_{x}}\left\{ \begin{array}{l} 2,\;\text{if}\;i=WW\\ 1,\;\text{if}\;i=Ww\\ 0,\;\text{if}\;i=ww \end{array}\right.\;\text{and},\;X_{a_{x}}\left\{ \begin{array}{l} 0,\;\text{if}\;i=WW\\ 1,\;\text{if}\;i=Ww\\ 0,\;\text{if}\;i=ww \end{array}\right.$$

Here, *Z* is the trait value, *C*_*k*_ is the random effect due to cohort, *F*_*l*_ is the effect of seed family (environmental maternal effect), which is nested within genotype; *a* and *d* are the single-locus effects, and *aa*, *ad*, *da*, and *dd* are the epistatic effects. The residual variance applies to variance within families. The *X*_*a*_ and *X*_*d*_ variables (corresponding to the design matrix in [18]) are numerical values that, together, specify an individual’s diploid genotype at a locus. In this case, *W* is the donor allele and *w* is the reference IM767 allele at a QTL. For a pair of loci, the four pairwise products of these variables provide contrasts by which the four analogous epistatic parameters are estimated. For a completely homozygous IM767 individual (*ww* at all loci), all *X*_*a*_ and *X*_*d*_ terms are 0, and therefore, the standard inbred IM767 line serves as the reference point in the *functional* NOIA model by which all genetic effects are defined as deviations from. These genetic effects can then be used (as described in the proceeding section) to generate predicted genotypic values for multi-locus genotypes. To determine the predicted genotypic values in the absence of epistasis (non-epistatic values), we fit separate models to estimate single-locus effects using only data corresponding to single-locus genotypes (essentially, the NIL genotype data that forms a subset of the DNIL data). Again, we use the *functional* NOIA parameterization (Eq 1 without epistatic terms), specifying the homozygous IM767 genotype as the reference point. By this method, we define the non-epistatic value as the predicted multi-locus genotypic value given only information from individual loci in an isogenic background.

It should be noted that the NOIA model has both a *statistical* formulation and a *functional* formulation, which is used here. Effectively, Eq 1 is the traditional animal model of genetic effects, and the *functional* NOIA (referred to as NOIA, hereafter) simply refers to the index variables used to specify an individual’s genotype. Here, we require functional genetic effects, in order to predict variances for any set of population allele frequencies. We investigated alternative, functional parameterizations for the index variables including the F_∞_ model [25] and the unweighted-regression (UWR) of Cheverud and Routman [15], but these models use a different reference point and the parameters have a different quantitative interpretation. F_∞_ and UWR yield the same predicted genotype values as NOIA (the models are inter-convertible), but we prefer the NOIA because parameters are defined as deviations from a reference genotype and, therefore, are more clearly interpretable between the full model (Eq 1) and the reduced (non-epistatic) model (fit to the reduced dataset). As a result, we find that the non-epistatic coefficients of NOIA (*a* and *d* terms of Eq 1) are “stable.” If we fit the full NOIA model (all terms) to the full dataset (all genotypic combinations included) we get estimates for the *a* and *d* terms that are nearly equivalent to what we get when we fit the reduced NOIA model (no interactions terms) to the reduced dataset (plants of the reference genotype plus those that differ from the reference genotype at only one QTL). This is not true of analogous estimates from the F_∞_ or UWR models, which do not use a common reference genotype as the reference point. While this consistency is convenient for the interpretation of genetic effects, it is not crucial to the results. It is the genotypic values predicted with and without epistasis that serve as the basis for determining the effect of epistasis on genetic variance, and as stated previously, the multiple parameterizations that we investigated all provide the same predicted values.

We used REML (implemented in JMP, Version *11*. SAS Institute Inc., Cary, NC, 1989–2014) to estimate the fixed genetic effects as well as accommodate random effects (cohort and family) in the model. While Eq 1 is specified for only 2 loci, all relevant genetic effects were included in the linear model and fit to the entire DNIL (full model) or NIL data (reduced model). Fitting this larger model accommodated the fact that many DNILs have overlapping genotypes. Models were fit separately for each trait. In total, there were 14 single-locus effects (seven ‘*a’* terms and seven ‘*d*’ terms) as well as 44 epistatic terms (four epistatic terms per DNIL x 11 DNILs). Model fits were based on 4263 measurements carried forward from Kelly and Mojica [3] plus 6234 measurements from the five additional grow-ups.

### From effects to variances

Estimation error in genetic effect estimates must be properly accommodated because genetic effect estimates are squared and different effects are multiplied together, when calculating variances. This can introduce bias with or without epistasis. Consider the single locus, 2-allele model [11], where the additive genetic variance (*V*_*A*_) is

$$V_{A}=2pq{\left[a+d(q-p)\right]}^{2}$$ (2)

Here, *a* and *d* are the additive effect and dominance deviation, respectively, and *p* and *q* are the frequencies of alternative alleles. An experimental study will yield estimates for the genetic effects, $\hat{a}$ and $\hat{d}$, but even if unbiased, these estimates will be encumbered with estimation error:

$$\hat{a}=a+\gamma_{a}$$ (3A)

$$\hat{d}=d+\gamma_{d}$$ (3B)

Here, the *γ* are residuals; random variables with mean 0 and a variance contingent on experimental design (e.g. sample sizes). If direct substitution is used to estimate *V*_*A*_, i.e. ${\hat{V}}_{A}=2pq{\left[\hat{a}+\hat{d}(q-p)\right]}^{2}$, bias is introduced:

$$E[{\hat{V}}_{A}]=2pq{\left[a+d(q-p)\right]}^{2}+2pq(Var[\gamma_{a}]+2(q-p)Cov[\gamma_{a},\gamma_{d}]+{\left(q-p\right)}^{2}Var[\gamma_{d}])$$ (4)

The second term of the sum, involving the estimation variances (*Var*[*γ*_*a*_], *Var*[*γ*_*d*_]) and the covariance of errors (*Cov*[*γ*_*a*_, *γ*_*d*_]), is the bias. A bias corrected estimate, denoted $V_{A}^{*}$, can be derived using standard dispersion statistics:

$$V_{A}^{*}={\hat{V}}_{A}-2pq(s_{a}^{2}+2(q-p)s_{ad}+{\left(q-p\right)}^{2}\;s_{d}^{2})$$ (5)

where $s_{a}^{2}$ is the estimated variance of *γ*_*a*_ (the squared standard error of $\hat{a}$), $s_{d}^{2}$ is the estimated variance of *γ*_*d*_ and *s*_*ad*_ is the sampling co-variance. This statistical issue has been addressed for a single locus [19], and we here generalize bias-correction for genetic variance predictions when there are interactions among loci. We extend the logic of Eq 5 to all eight genetic effect estimates associated with each QTL pair (see “Linear model for estimation of genetic effects” section above).

### Prediction of variance components

The genetic variance of a trait for a particular population is a function of the individual genotypic values and their frequency in the population. For a particular multi-locus genotype (*u*), we can calculate the predicted genotypic value using the genetic effect estimates from the linear model fit as follows:

$${\hat{Z}}_{G,u}=\sum_{i=1}^{B}{\hat{b}}_{i}X_{i,u}$$ (6)

where ${\hat{b}}_{i}$ is the estimate for the i’th effect (*B* is the total number of effects), and *X*_*i*,*u*_ is the relevant indicator variable from Eq 1 for genotype *u*. If we let *Z*_*G*_ represent the genotypic value of an individual drawn randomly from a population, then the total genetic variance, *V*_*G*_, is simply the variance of *Z*_*G*_, which can be found by,

$${\hat{V}}_{G}=Var[{\hat{Z}}_{G}]=E[{\hat{Z}}_{G}^{2}]-E{\left[{\hat{Z}}_{G}\right]}^{2}$$ (7)

These expected values are functions of the genotypic values and the multi-locus genotype frequencies. For example,

$$E[{\hat{Z}}_{G}^{2}]=\sum_{u\in \Omega }F_{u}{\hat{Z}}_{G,u}^{2}$$ (8)

where Ω is the set of all possible multi-locus genotypes and *F*_*u*_ is the population frequency of the multi-locus genotype, *u*. Expanding ${\hat{Z}}_{G}^{2}$ (temporarily suppressing the u subscript), we see that

$${\hat{Z}}_{G}^{2}=(X_{1}{\hat{b}}_{1}+X_{2}{\hat{b}}_{2}\ldots )(X_{1}{\hat{b}}_{1}+X_{2}{\hat{b}}_{2}\ldots )=X_{1}^{2}{\hat{b}}_{1}^{2}+X_{1}X_{2}{\hat{b}}_{1}{\hat{b}}_{2}+\ldots$$ (9)

We see that ${\hat{Z}}_{G}^{2}$ is biased because $E[{\hat{b}}_{i}^{2}]>b_{i}^{2}$ and $E[{\hat{b}}_{i}{\hat{b}}_{j}]\neq b_{i}b_{j}$, essentially the same reason evident in Eq 4 We correct for this upward bias by subtracting off the relevant sampling variance/covariance term, such that the corrected value is,

$$Z_{G}^{2}{}^{*}=X_{1}^{2}b_{1}^{2}+X_{1}X_{2}b_{1}b_{2}+\ldots =X_{1}^{2}({\hat{b}}_{1}^{2}-s_{{\hat{b}}_{1}}^{2})+X_{1}X_{2}({\hat{b}}_{1}{\hat{b}}_{2}-s_{{\hat{b}}_{1}{\hat{b}}_{2}})+\ldots$$ (10)

More generally,

$$Z_{G}^{2}{}^{*}=\sum_{i=1}^{B}\sum_{k=1}^{B}X_{i}X_{k}({\hat{b}}_{i}{\hat{b}}_{k}-s_{{\hat{b}}_{i}{\hat{b}}_{k}})$$ (11)

noting that $s_{{\hat{b}}_{i}{\hat{b}}_{k}}$ is equal to $s_{{\hat{b}}_{i}}^{2}$. Thus, the expected value for Eq 11 is

$$E[Z_{G}^{2*}]=\sum_{u\in \Omega }F_{u}\sum_{i=1}^{B}\sum_{k=1}^{B}X_{u,i}X_{u,k}b_{i}b_{k}$$ (12)

Correcting the estimate for *E*[*Z*_*G*_]^2^ is slightly more involved because the full expansion (substituting Eq 6 for *Z*_*G*_) produces terms of squares and cross-products within and across multi-locus genotypes. The bias corrected estimator for *E*[*Z*_*G*_]^2^ is

$$\sum_{u\in \Omega }\sum_{v\in \Omega }F_{u}F_{v}\sum_{i=1}^{B}\sum_{k=1}^{B}X_{u,i}X_{v,k}(b_{i}b_{k}-s_{{\hat{b}}_{i}}s_{{\hat{b}}_{k}})$$ (13)

The additive genetic variance, *V*_*A*_, due to a set of *L* bi-allelic loci is

$$V_{A}=\sum_{k=1}^{L}2p_{k}q_{k}\alpha_{k}^{2}$$ (14)

where *α*_*k*_ is the average effect of the allele with frequency *p*_*k*_ at locus *k*. The average effect is *α*_*k*_ = *α*_*k*,1_ – *α*_*k*,2_, where

$$\alpha_{k,1}=p_{k}E[Z|W_{k}W_{k}]+q_{k}E[Z|W_{k}w_{k}]$$ (15)

and

$$\alpha_{k,2}=p_{k}E[Z|W_{k}w_{k}]+q_{k}E[Z|w_{k}w_{k}]$$ (16)

Here, *E*[*Z* | *W*_*k*_ *W*_*k*_] is the mean phenotype across all multi-locus genotypes that are homozygous for allele 1 at locus k, *E*[*Z* | *W*_*k*_ *w*_*k*_] is the corresponding conditional mean for heterozygotes, and *E*[*Z* | *w*_*k*_ *w*_*k*_] is the mean for allele 2 homozygotes. Eqs 14–16 assume Hardy-Weinberg genotype proportions. We focus on the Hardy-Weinberg case, because without random mating, the additive genetic variance is not a sufficient statistic to predict response to selection [26–28].

The average effect is a linear function of genetic effects, and as a consequence, direct substitution of effect estimates into Eqs 14 and 15 yields unbiased estimates. However, when *α*_k_ is squared (Eq 13), upward bias is introduced by estimation error. Computation of bias-corrected estimates *α*_*k*_^2^ follows the same method of Eqs 7–13, although the relevant sums are over all loci except *k* (code to implement these calculations was written in C; available in supplemental information). Importantly, in these calculations, we assume that epistasis is absent for pairs of QTL corresponding to DNILs for which we have no measurements. We also assume no higher-order interactions. All parameter estimates from the linear model fit were incorporated regardless of their statistical significance. Our method accounts for uncertainty in parameter estimates by directly incorporating the sampling variance/covariance of estimates into the calculation of variance components. Predictions of *V*_*G*_, *V*_*A*_, and locus specific *α*_*k*_ were generated for each set of simulated allele frequencies.

### Allele frequency model

We investigated the distributions of genetic variance components under two differing allele frequency models, a uniform distribution and a U-shaped distribution as in Hill *et al*. [12]. Allele frequencies were sampled independently for each locus to create a set of 7 frequencies per set. We drew 200 sets of frequencies from each distribution, and these have been included in the supplemental information. For each set of allele frequencies and for each trait, we calculated the corrected and uncorrected genetic variance components variance (Eqs 6–16). We performed this operation, first, using the entire suite of single-locus and epistatic genetic effects to predict genotypic values, and then a second time, using only the single-locus genetic effects estimated from the reduced data set consisting only of single-locus genotypic data. This allows us to observe the effect of epistasis on the total and locus-specific genetic variance components.

### A simulation test of the bias-correction procedure

The bias-correction procedure will produce unbiased estimates of variance components if the estimated sampling (co)variances ($s_{{\hat{b}}_{i}{\hat{b}}_{j}}$) are unbiased estimates of the true sampling (co)variances. However, precision of bias-corrected estimates may be reduced, as including $s_{{\hat{b}}_{i}{\hat{b}}_{j}}$ in the calculations could increase sampling variance of bias-corrected estimates, particularly if $s_{{\hat{b}}_{i}{\hat{b}}_{j}}$ terms are large. We performed parametric bootstrapping to determine the effect of our procedure on the precision of estimates as well as to confirm its efficacy in eliminating bias. Using the estimated genetic effects as well as the within-group variance estimated from the linear model fits, we simulated trait values for individuals of particular genotypes producing 500 replicate data sets. Each simulated dataset for each trait was the same size and structure as the original dataset. We then estimated the genetic effects and sampling (co)variance matrices for each replicate dataset and calculated the corrected and uncorrected genetic variance components for two sets of allele frequencies. For the first set, the reference allele frequency was set equal to 0.5 for all loci. For the second set, the reference allele frequency was set equal to 0.05, and thus, the donor allele frequency was set to 0.95 (see “Linear model for estimation of genetic effects” section above for definition of reference vs. donor). To determine the bias exhibited by each variance calculation, we calculated the true variances given the genetic effects that we specified to simulate the data. We calculated the mean square error for each distribution of variance calculations by dividing the sum of squared deviations from the true value by the number of replicates (500). To standardize the mean square error, we divided the sum by the square of the true variance. In addition, we calculated the standardized bias as $\frac{x^{*}-x}{x}$, where x* is the average of estimates and *x* is the true value.

## Results

### Prevalence and patterns of epistasis between QTL

**Fig 1:**
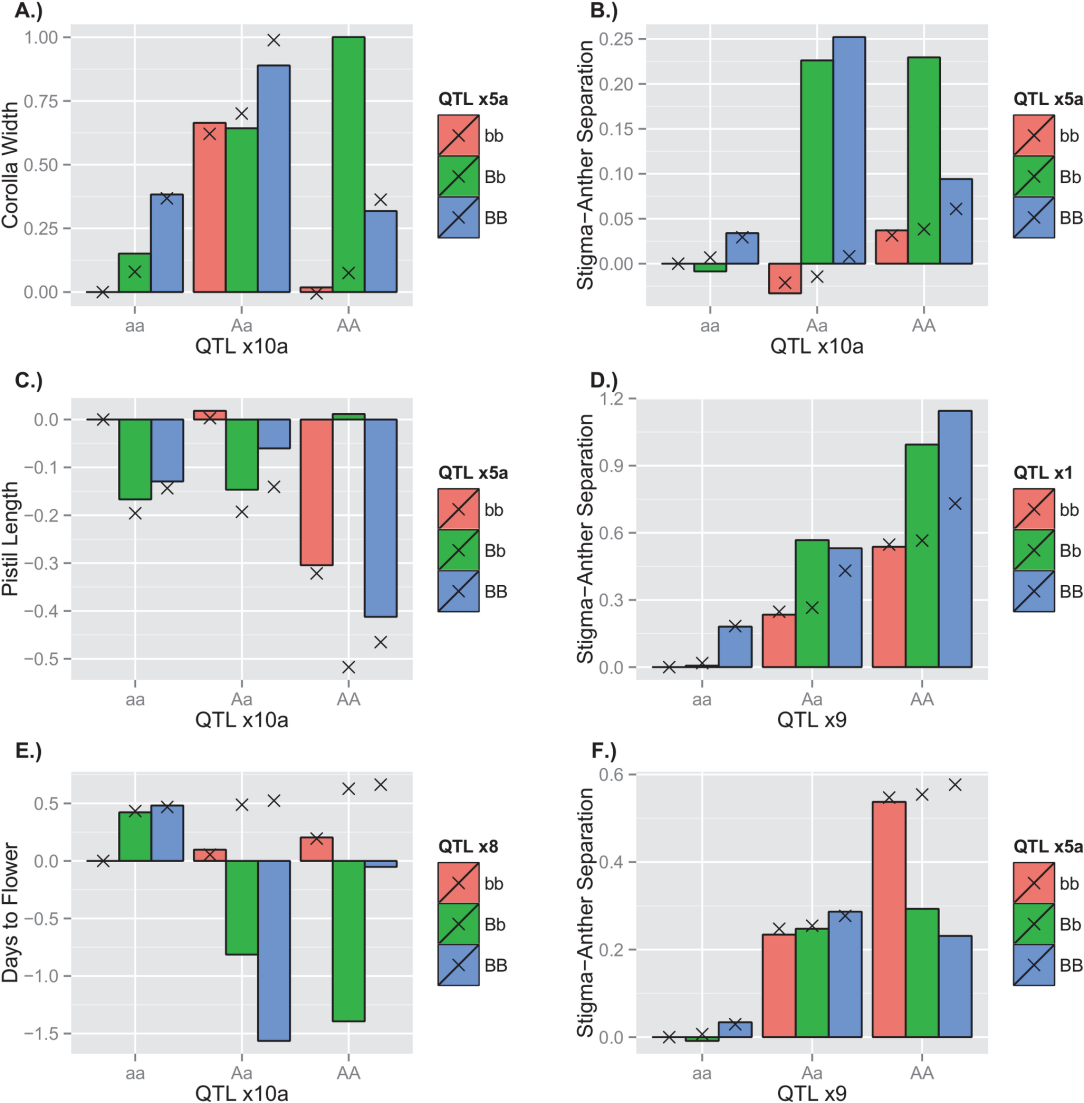
Trait values (in mm’s) for selected DNILs as deviations from the reference genotype (IM767) estimated by the linear model. Genotypic mean values are given by bars, whereas non-epistatic values are given by the X’s. A.) Corolla Width for DNIL x10a —x5a; B.) Stigma-Anther (SA) Separation for DNIL x10a —x5a; C.) Pistil Length for DNIL x10a —x5a; D.) SA Separation for DNIL x9—x1; E.) Days to Flower for DNIL x10a —x8; F.) SA Separation for DNIL x9—x5a.

There is strong evidence for epistasis for all of the traits (Table 1), consistent with the prior study of these loci based solely on homozygous genotypes [3]. Concerning the individual terms of the models, we find that epistatic genetic effects are occasionally significant and typically of the same order as single-locus effects (S2 Table and S3 Table). There was no clear trend towards positive or negative epistasis, although additive-by-additive interactions are more frequently observed to be significant than other forms. Particular types of epistasis are illustrated by example in Fig 1 (see S1 Fig for the full collection of graphs), which contrast the predicted genotypic value with epistasis (the bars) to the corresponding non-epistatic value (the ‘X’). The non-epistatic value is the genotypic value predicted using only the relevant single-locus effects (*a* and *d* terms) estimated from a linear model fit based on single-locus genotype data (essentially, NILs). The epistatic genotypic value is based on all genetic effects (single-locus and epistatic) estimated from the full linear model fit to the DNIL data. The deviation between these values is the contribution of epistasis to the observed genotypic value. Fig 1E provides an example of sign epistasis, wherein the positive effect of the donor allele at QTL *x8* in the QTL *x10a* AA background exhibits a negative effect in the Aa background. Fig 1A–1C demonstrate the potential for consistent epistatic deviations for certain genotypes across multiple traits; particularly, the AABb genotype exhibits striking positive epistasis for all morphological traits. Conversely, some genetic backgrounds have variable effects when combined with other loci. For instance, positive synergism is observed for QTL *x1* in the QTL *x9* AA background (Fig 1D), whereas negative diminishing returns epistasis is observed for QTL *x5a* in the same AA background of QTL *x9*. Lastly, Fig 1A–1C also provides evidence of the potential of epistasis to modify dominance relationships. In the AA background, overdominance emerges for all traits, despite single-locus predictions of partial dominance (Fig 1A and 1B) and underdominance (Fig 1C).

**Table 1:** Model comparison for models with (Full) and without (Reduced) epistasis terms included.

Trait	Model	AIC	LogLike	*X* ^*2*^	P-value
Pist	Reduced	31463	-15713	87.433	0.0002
Pist	Full	31464	-15669		
SA	Reduced	20613	-10288	146.3	<0.0001
SA	Full	20555	-10215		
CW	Reduced	42092	-21027	80.123	0.0007
CW	Full	42099	-20987		
DTF	Reduced	52782	-26372	65.823	0.0182
DTF	Full	52804	-26339		

The degrees of freedom for each test was 44.

When considered together, the epistatic deviations (deviation between the ‘X’s and bars in Fig 1) illustrate the pattern of epistasis. Focusing on only the genotypes unobserved in the single-locus NIL model fit (AABB, AaBB, AABb, and AaBb), we find that the average epistatic deviation is near zero for corolla width, SA separation, and pistil length (-0.04, 0.01, and 0.06 respectively), but is more appreciable for days to flower (-0.49). This indicates that plants tended to flower earlier than expected based on non-epistatic predictions. The standard deviations of the epistatic deviations speaks to the variability of epistatic effects and are 0.43, 0.20, 0.30, and 0.68 for corolla width, SA separation, pistil length, and days to flower, respectively. Comparing the sum of absolute deviations from the reference genotype for non-epistatic versus epistatic values provides additional information on the pattern of epistasis. If we subtract the sum of epistasis values from the sum of non-epistatic values, a negative difference would indicate synergism (sometimes called positive epistasis), wherein epistasis gives rise to greater deviations from the reference point, on average. The converse would indicate a diminishing effect of mutations under epistasis. For corolla width, days to flower, pistil length, and SA separation, the percent difference between the sum of absolute deviations for no-epistasis vs. epistasis was 0.04, 0.02, -0.11, and 0.01, respectively. Evidence for cumulative synergism or diminishing returns is rather weak for all cases.

### Pleiotropy and correlation among traits

Pleiotropy is common for both single-locus and epistatic effects (S2 Table and S3 Table). Effects were typically significant for between two and three traits. Pleiotropy seems to be modular in the sense that QTL/DNILs with a significant effect on one floral trait tends to also affect other floral traits, in contrast to the day of flowering. Effects were significantly correlated between pistil length and corolla width (r = 0.47; p = 0.0002), between pistil length and days to flower (r = -0.28, p = 0.0305), and between pistil length and stigma-anther separation (r = 0.29, p = 0.0292; S4 Table for full list of values). The traits themselves were also strongly correlated (S5 Table). While some of the effects are in line with the correlational structure of the data (e.g. single-locus additive effect of QTL *x10b*), this was not a consistent pattern (e.g. single-locus additive effect of QTL *x5a*).

### Extension of genetic effects to genetic variance predictions

**Fig 2:**
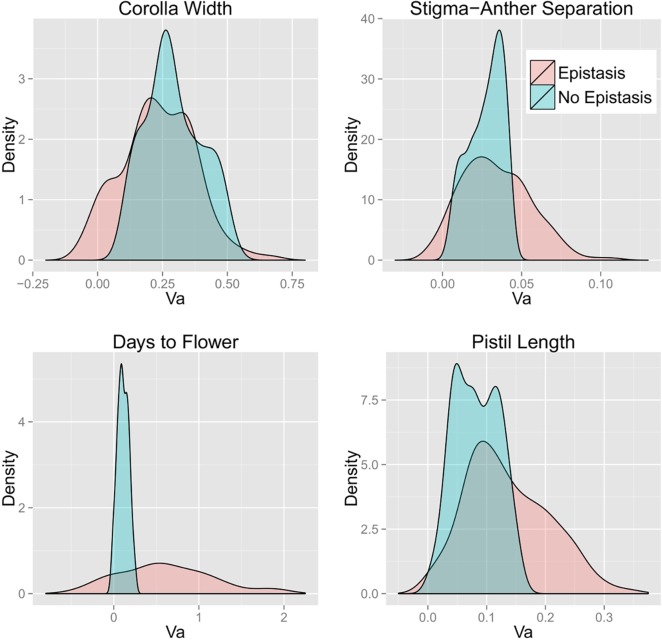
Density plots of bias-corrected *V*_*A*_ calculated with and without epistasis for each trait. Allele frequencies were drawn from a uniform distribution.

Density plots of *V*_*A*_ across the 200 simulated allele frequency sets (uniform distribution) calculated from the single-locus (termed “No Epistasis”) and DNIL (“Epistasis”) data are depicted in Fig 2 (the corresponding densities for the U-shaped allele frequency distribution are given as S4 Fig). With the exception of Corolla Width, epistasis produced an increase in the average of both *V*_*A*_ and *V*_*G*_ for all traits (Table 2) regardless of the allele frequency distribution. As expected, average *V*_*A*_ and *V*_*G*_ are always larger for Uniform distribution compared to the U-shaped distribution, whereas the proportion of variance that is additive is greater for the U-shaped distribution. Notably, the genetic variance is mostly additive even in the presence of substantial epistatic interactions. Epistasis also significantly affected the shape (variance) of the distributions of genetic variance estimates in addition to the location (mean). This is evident in Fig 2, as well as S4–S10 Figs. Epistasis tended to increase the variance, oftentimes producing long tails representing the observance of more extreme values.

**Table 2:** Mean and standard deviation (in parentheses) for additive and total genetic variance calculated with and without bias-correction (above and below the line, respectively), as well as with and without epistasis for both of the allele frequency distributions.

		Uniform distribution		U-Shaped distribution	
		Epistasis	No Epistasis	Epistasis	No Epistasis
DTF	Va	0.62 (0.57)	0.11 (0.07)	0.38 (0.51)	0.04 (0.07)
	Vg	0.99 (0.68)	0.01 (0.05)	0.67 (0.73)	-0.01 (0.06)
	Va/Vg	0.6	—	0.57	—
CW	Va	0.23 (0.14)	0.29 (0.11)	0.16 (0.13)	0.18 (0.1)
	Vg	0.36 (0.13)	0.41 (0.12)	0.21 (0.16)	0.23 (0.12)
	Va/Vg	0.64	0.71	0.74	0.77
SA	Va	0.03 (0.02)	0.03 (0.01)	0.02 (0.02)	0.02 (0.01)
	Vg	0.06 (0.02)	0.03 (0.01)	0.03 (0.03)	0.01 (0.01)
	Va/Vg	0.61	1.05	0.78	1.04
Pist	Va	0.14 (0.07)	0.08 (0.04)	0.09 (0.07)	0.05 (0.03)
	Vg	0.17 (0.07)	0.11 (0.04)	0.11 (0.08)	0.06 (0.04)
	Va/Vg	0.80	0.78	0.82	0.82
DTF	Va	1.19 (0.57)	0.25 (0.06)	0.79 (0.55)	0.14 (0.08)
	Vg	2.51 (0.95)	0.28 (0.06)	1.47 (0.95)	0.15 (0.08)
	Va/Vg	0.47	0.9	0.54	0.92
CW	Va	0.37 (0.14)	0.32 (0.11)	0.25 (0.13)	0.2 (0.1)
	Vg	0.74 (0.17)	0.47 (0.13)	0.41 (0.19)	0.27 (0.13)
	Va/Vg	0.50	0.68	0.63	0.75
SA	Va	0.05 (0.02)	0.03 (0.01)	0.03 (0.02)	0.02 (0.01)
	Vg	0.1 (0.03)	0.03 (0.01)	0.05 (0.03)	0.02 (0.01)
	Va/Vg	0.50	0.93	0.66	0.94
Pist	Va	0.19 (0.08)	0.09 (0.04)	0.13 (0.08)	0.06 (0.03)
	Vg	0.32 (0.1)	0.13 (0.04)	0.19 (0.1)	0.08 (0.04)
	Va/Vg	0.59	0.73	0.69	0.78

The bias-correction procedure significantly reduced estimated *V*_*G*_ values (particularly for days to flower; DTF) although there was considerable variation among sets (Table 2). The larger reduction due to bias-correction of DTF is expected given that estimation error is greatest for this trait and that this estimation error is directly related to the degree of bias in variance calculations. Bias-correction also reduced predicted *V*_*A*_, but to a lesser extent. As a consequence, the ratio of *V*_*A*_ to *V*_*G*_ is substantially greater for bias-corrected values (60–80% across the four traits) than for uncorrected variance components (45–60%). This is true regardless of whether one calculates *V*_*A*_/*V*_*G*_ for each simulation replicate and then averages, or takes the ratio of mean *V*_*A*_ to mean *V*_*G*_ (as in Hill *et al*. [12]). Occasionally, unrealistic, negative values for *V*_*A*_ result from the bias-correction procedure, and this tendency seems to be exacerbated by greater estimation error of the coefficient estimates. For example, effect estimates for Days to Flower routinely had the largest standard errors, and this is accompanied by many negative values (Fig 2, lower left panel). It should be noted that uncorrected variance estimates also produce negative values albeit to a lesser extent (Figs S8 and S10), which is true for any estimate whose standard error is large relative to its magnitude.

The distributions resulting from the bias-correction simulations demonstrate that the correction procedure is effective (Table 3 and Figs S11 and S12). The mean of corrected estimates matches the truth more closely than uncorrected estimates (Std. Bias in Table 3). There is a slight negative bias to the bias corrected estimate (typically 1–4%), perhaps because the sampling (co)variances of estimates are only approximate. The mean square error of corrected statistics is lower than for the uncorrected (Table 3). This is due entirely to the bias reductions given that the distributions of the corrected and uncorrected statistics have nearly identical variances.

**Table 3:** Standardized mean square error (MSE) and standardized bias for corrected (Corr.) and uncorrected (Unc.) variance estimates from the bias-correction simulations.

		q = 0.5				q = 0.05			
		MSE		Std. Bias		MSE		Std. Bias	
		Corr.	Unc.	Corr.	Unc.	Corr.	Unc.	Corr.	Unc.
DTF	Vg	0.07	0.12	-0.04	0.35	0.17	0.24	0.00	0.26
	Va	0.12	0.12	-0.04	0.33	0.23	0.31	0.01	0.28
CW	Vg	0.07	0.15	-0.01	0.27	0.27	0.39	-0.04	0.35
	Va	0.07	0.09	-0.01	0.13	0.30	0.41	-0.04	0.33
SA	Vg	0.10	0.18	-0.02	0.27	0.51	1.16	-0.03	0.76
	Va	0.18	0.24	0.00	0.20	0.79	1.38	-0.04	0.80
Pist	Vg	0.06	0.11	-0.03	0.23	0.17	0.33	-0.02	0.40
	Va	0.09	0.09	-0.03	0.07	0.06	0.10	-0.03	0.41

**Fig 3:**
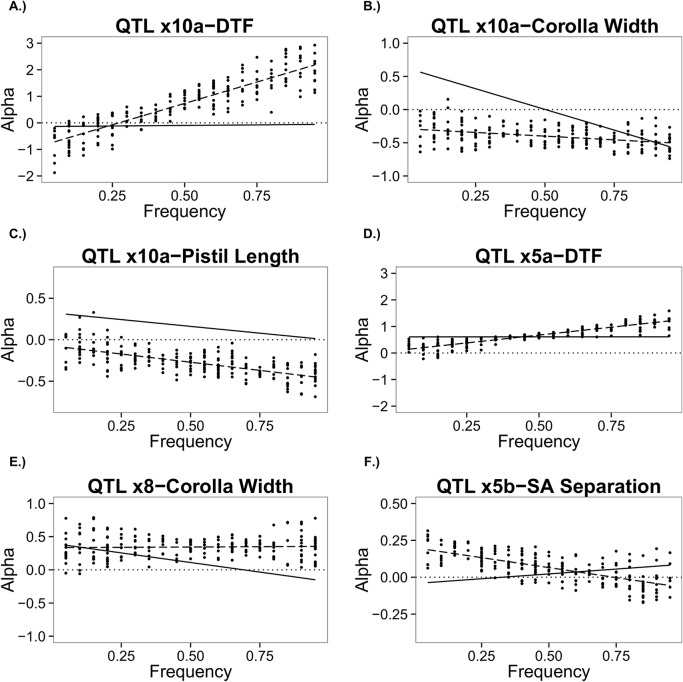
Average effect (Alpha) values for a QTL plotted against frequency of the QTL. The solid black line indicates the alpha value calculated without epistasis whereas the dashed line shows the best-fit line through the scatter of points, which are the alpha values calculated with epistasis included. A.) Days to Flower for QTL x10a; B.) Corolla Width for QTL x10a; C.) Pistil Length for QTL x10a; D.) Days to Flower for QTL x5a; E.) Corolla Width for QTL x8; F.) Stigma-Anther Separation for QTL x5b.

The *V*_*A*_ for traits is a weighted sum of squared average effects (Eqs 14–16). When considering the effects of epistasis on individual loci, we see dependence of the average effect, α, on genetic background (Fig 3 for selected examples, S2 Fig for full collection). The points in the figure are α values for a locus calculated from our set of 200 allele frequencies (uniform distribution), and the dashed line represents the best-fit line through the points. The solid black line shows the values for α without epistasis, which depend only on the genetic effects and allele frequency at that locus. If the locus showed entirely additive gene action, this line would be perfectly horizontal, whereas dominance gives rise to a non-zero slope. Over- or under-dominance is implied when lines cross 0 on the y-axis. Deviations between the dashed and solid lines in Fig 3 demonstrate the effect of epistasis averaged over genetic backgrounds. A change in slope between solid and dashed lines indicates the statistical dominance effect depends on epistasis. In Fig 3A, we see that locus QTL *x10a* is predicted to contribute little to no *V*_*A*_ without epistasis, but exhibits a substantial average effect when epistasis is considered. Fig 3C indicates a case in which epistasis does not affect dominance, but changes the sign of α. However, epistasis often affects the dominance properties of a locus as evidenced by differences in the slope of the dashed and solid lines. Epistasis is seen to make an additive locus exhibit dominance (Fig 3A and 3D), reduce dominance to near additivity (Fig 3B and 3E), and give rise to over or underdominance (Fig 3A and 3F). Some loci are relatively less sensitive (more robust) to background than others: Note the small scatter and relatively shallow trajectory of *x5a* relative to *x10a* for Days to Flower (Fig 3A and 3D).

## Discussion

Epistasis is often a major factor in the mapping from genotype to phenotype [2, 5, 29, 30], but its relevance to heritability and evolution remains contentious [9, 10]. In part, this is due to persistent confusion about how genetic effects are defined and, thus, relate to genetic variation. Kempthorne [31] and Cockerham [32] defined genetic effects to be orthogonal, such that additive, dominance, and epistatic variance were each attributed solely to the corresponding genetic effects. The orthogonal property of these models is convenient, and the resulting estimates relate directly to evolutionary change. However, the estimated effects are specific to the population under consideration and depend entirely on allele frequencies. Epistatic effects defined in this way do not contribute to the additive variance (*V*_*A*_), despite that interactions between genes certainly do affect *V*_*A*_. This has led many to the incorrect assumption that epistasis is unimportant for evolution. Functional models provide an alternative view that better reflect the physiological or molecular interactions that determine the mapping from genotype to phenotype [15]. A substantial body of theory and simulation has shown that gene interactions can be an important determinant of heritable variation and, thus, the response to selection [14, 16, 30].

Unfortunately, key empirical evidence about how epistasis influences heritability is lacking. Most basically, do interactions among genes tend to increase or decrease *V*_*A*_ on average? How does epistasis affect the evolution of *V*_*A*_ under sustained selection? How do interactions among QTLs affect the allele frequency dynamics that underpin changes in quantitative traits? While there are many empirical studies demonstrating gene interactions, nearly all lack a clear population context and heritability is a population-specific statistic. While crosses between divergent populations/species routinely reveal epistasis, the loci segregating in these crosses may never have been segregating (simultaneously) within a specific natural population (e.g. Dobzansky-Muller incompatibilities). More importantly, the magnitude and pattern of effects may not be representative of the segregating polymorphisms that determine genetic variation within populations, perhaps due to multiple, subsequent mutation in a locus [33] (see Hansen [34] for a discussion on the evolution of epistatic interactions). By focusing on epistasis between loci polymorphic in a single, natural population of *M*. *guttatus*, we provide an empirically calibrated evaluation of how gene interactions alter genetic variance components in nature. We find that epistasis has a net positive effect on both *V*_*A*_ and *V*_*G*_ for most measured traits, providing an empirical demonstration of epistasis in determining heritable variation and the response to selection.

The contribution of epistasis to genetic variance depends, in part, on the existence of particular patterns of epistasis among loci [14, 35]. Synergistic epistasis among mutations, termed positive epistasis by [14] (but see [36]), will increase variability among genotypes, whereas diminishing returns epistasis should have the opposite effect. However, when interactions are variable and idiosyncratic as we observe in this study, the effect on genetic variance will likely depend more on allele frequencies in the population than any average pattern across loci. As allele frequencies change, the balance between positive and negative effects of gene interactions on genetic variance also changes in proportion to the relative frequency of different genotypic combinations. Indeed, we routinely observe both positive and negative effects in the genetic variance predictions for all traits across simulated allele frequency sets (Fig 2). Epistasis will thus facilitate evolution in some cases, but hinder it in others. It may do both at different points in time within the same evolving population.

The average tendency for epistasis to increase genetic variance for days to flower, pistil length, and SA separation suggest a positive average effect of interactions on the additive genetic variance. The exception is corolla width where the mean *V*_*A*_ is slightly lower with epistasis; a result that could be anticipated from the observation in Kelly and Mojica [3] that corolla width QTLs had smaller effects in combination than predicted from individual effects in the isogenic background. For all traits, epistasis increased the variance of the *V*_*A*_ across allele frequency sets (Fig 2 and Table 2). This speaks directly to how *V*_*A*_ will change under directional selection. With only additive effects, *V*_*A*_ is predicted to change slowly with selection, unless there are major loci and/or if allele frequencies are initially extreme. The greater dispersion of *V*_*A*_ with interactions (Fig 2) suggests that epistasis is likely to generally accelerate changes in *V*_*A*_.

The strong dependency of the average effect of substitution at a locus on interactions with other genotypes (Fig 3) indicates that epistasis also has important implications for the dynamics of individual loci. With directional selection, the change in allele frequency at a QTL is determined by the locus-specific *V*_*A*_, which, in turn, is proportional to the average effect [37, 38]. Our results suggest that average effects and, consequently, locus-specific *V*_*A*_ will be highly malleable throughout the selection response as allele frequencies change simultaneously across all loci. This is an important consideration now that we are able to directly monitor allele frequency change within populations under sustained directional selection [39–44]. Allele frequency trajectories will be variable in finite populations, and epistasis is likely to amplify this variability, generating idiosyncratic responses to replicate selection events [42, 45–47]. Even when genetic drift is inconsequential, sign epistasis and emergent over/under dominance imply that the ultimate loss, fixation, or balance of an allele will depend on the order of fixation of alleles at other loci. A thorough characterization of epistasis is, therefore, necessary if one wishes to understand allelic dynamics in response to selection and how this translates to the observed change in trait means.

Characterizing higher order epistastic interactions involving three or more loci may be necessary for a complete understanding of the selection response in terms of the underlying loci. Nevertheless, studies of pairwise epistasis like this one provide important information on the relative role of at least a subset of possible interactions. First, they provide a baseline estimate of the genetic variance attributable to epistasis. Second, they provide insight regarding the interactive properties of a locus (i.e. how frequently and strongly does the locus exhibit epistasis, and to what degree does this influence the average effect at the locus). Lastly, pairwise estimates allow us to determine the improvement in predictive ability of the selection response due to the inclusion of epistasis. For example, Carlborg *et al*. [48] demonstrate that pairwise estimates of epistasis are necessary to predict the long-term selection response in an artificial selection experiment on growth in domestic chickens. While only a first step towards understanding the entire network of epistatic interactions, pairwise estimates illustrate the relevance of epistasis to heritable variation and the evolutionary process.

To extend from genetic effects to genetic variances, it is essential to accommodate estimation error. Otherwise, predictions will necessarily be upwardly biased. Though this issue has been addressed previously [19], it has gone unnoticed in the vast majority of studies extending genetic effects to variances. Even in studies utilizing highly replicated measurements from inbred lines, such as this one, estimation error is appreciable, and the greater the estimation error, the greater the upward bias in variance predictions. This is particularly disconcerting given the disproportionate effects on variance components documented in this study, as this will impact estimates of heritability in the narrow sense. We have shown that our method of correcting variance calculations does indeed remove this bias and remains as precise as uncorrected calculations, although the latter point is likely to depend on the sample size of a study and the inherent variation of a particular trait. In smaller studies, one must make the choice between the tradeoff of a biased estimate vs. an imprecise one. However, we stress that future studies attempting to unite genetic effects with heritability take greater care to accommodate the potential for estimation error to inflate estimates of genetic variance components.

### Conclusion

The results of this study, documenting the role of variable epistasis in determining genetic variance components are timely given the renewed interest and debate on the subject [9, 10]. Given that most genetic variance remains additive in the presence of epistasis and that additive variance is largely sufficient to predict the response to selection, it would seem, at first glance, as if epistasis is irrelevant. Upon further inspection, we find that epistasis contributes substantially to additive genetic variance, increasing it on average for most traits (Table 2 and Fig 2), which should accelerate the response to selection. We note however that epistasis reduces the additive variance for particular combinations of allele frequencies with all traits. Contrary to the perspective that epistasis will have only transient effects on selection dynamics due to allelic combinations held together by linkage disequilibrium [37], our results suggest that the principal effect of epistasis may be as a major determinant of *V*_*A*_ [10, 14], although empirical evidence supporting the generality of this conclusion is currently limited [15].

Semantics has been a major impediment to connecting epistasis and the additive variance; the terms additive, dominance, and epistatic effects are used in a broad range of genetic effect models, yet differ substantially both in their interpretation as well as their relationship to genetic variance. This has led several authors to make general statements regarding epistasis that may be valid in the context of their own study, but are incorrect generally. For instance, it is not uncommon for authors to simply claim that non-additive effects, such as dominance and epistasis, do not contribute to additive genetic variance [49] and, therefore, are unimportant for the evolution of polygenic traits [9]. While this is true of statistical models of genetic effects, it is not true of functional models, as this and several other articles have argued [10, 14, 15]. In addition to partially determining additive variance, epistasis implies that allelic dynamics will depend on initial frequencies, such that replicate selection events are expected to be largely idiosyncratic and perhaps unrepeatable in terms of changes at underlying loci. Therefore, understanding functional epistastic interactions are important for understanding the fate of individual genes as well as populations exposed to selection.

## Supporting Information

S1 Data Phenotype date used for estimation of genetic effects as well as sampling (co)variance matrices. (XLSX)

S1 Code C code used to calculate variance components. (C)

S1 Table Marker names and linkage group for each QTL of the DNIL line sets. (DOCX)

S2 Table Single-locus effect estimates for each QTL on each trait. * = 0.1 > p > 0.01; ** = 0.01 > p >0.001; *** = p < 0.001.
(DOCX)

S3 Table Epistatic effect estimates for all DNILs. * = 0.1 > p > 0.01; ** = 0.01 > p >0.001; *** = p < 0.001.
(DOCX)

S4 Table Pairwise correlations between the effect estimates for the different traits. (DOCX)

S5 Table Pairwise correlations between traits. (DOCX)

S1 Fig Genotypic means and non-epistatic values for DNILs. (DOCX)

S2 Fig α values for each QTL plotted against frequency of the QTL from the Uniform distribution. The black line indicates the alpha value calculated without epistasis whereas the blue line shows the best-fit line through the scatter of points, which are the alpha values calculated with epistasis included.
(DOCX)

S3 Fig α values for each QTL plotted against frequency of the QTL from the U-shaped distribution. The black line indicates the alpha value calculated without epistasis whereas the blue line shows the best-fit line through the scatter of points, which are the alpha values calculated with epistasis included.
(DOCX)

S4 Fig Distributions for corrected additive genetic variance for the U-shaped distribution of allele frequencies. (DOCX)

S5 Fig Distributions for corrected genetic variance for the Uniform distribution of allele frequencies. (DOCX)

S6 Fig Distributions for corrected genetic variance for the U-shaped distribution of allele frequencies. (DOCX)

S7 Fig Distributions for uncorrected genetic variance for the Uniform distribution of allele frequencies. (DOCX)

S8 Fig Distributions for uncorrected genetic variance for the U-shaped distribution of allele frequencies. (DOCX)

S9 Fig Distributions for uncorrected additive genetic variance for the Uniform distribution of allele frequencies. (DOCX)

S10 Fig Distributions for uncorrected additive genetic variance for the U-shaped distribution of allele frequencies. (DOCX)

S11 Fig Distributions for additive genetic variances calculated with and without bias-correction for the bias-correction simulations. The black arrows indicate the ‘true’ value predicted using the effects from which data was simulated.
(DOCX)

S12 Fig Distributions for genetic variances calculated with and without bias-correction for the bias-correction simulations. The black arrows indicate the ‘true’ value predicted using the effects from which data was simulated.
(DOCX)

## References

[1] Carlborg Ö., et al, A global search reveals epistatic interaction between QTL for early growth in the chicken. Genome research, 2003 13(3): p. 413–421. 12618372 10.1101/gr.528003 PMC430275

[2] Huang W., et al, Epistasis dominates the genetic architecture of Drosophila quantitative traits. Proceedings of the National Academy of Sciences, 2012 109(39): p. 15553–15559. 22949659 10.1073/pnas.1213423109 PMC3465439

[3] Kelly J.K. and Mojica J.P., Interactions among flower-size QTL of Mimulus guttatus are abundant but highly variable in nature. Genetics, 2011 189(4): p. 1461–1471. 10.1534/genetics.111.132423 21926295 PMC3241418

[4] Li Z., et al, Epistasis for three grain yield components in rice (Oryxa sativa L.). Genetics, 1997 145(2): p. 453–465. 9071598 10.1093/genetics/145.2.453 PMC1207809

[5] Moore J.H., The ubiquitous nature of epistasis in determining susceptibility to common human diseases. Human heredity, 2003 56(1–3): p. 73–82. 14614241 10.1159/000073735

[6] Shimomura K., et al, Genome-wide epistatic interaction analysis reveals complex genetic determinants of circadian behavior in mice. Genome research, 2001 11(6): p. 959–980. 11381025 10.1101/gr.171601

[7] Zuk O., et al, The mystery of missing heritability: Genetic interactions create phantom heritability. Proceedings of the National Academy of Sciences, 2012 109(4): p. 1193–1198. 10.1073/pnas.1119675109 22223662 PMC3268279

[8] Bloom J.S., et al, Finding the sources of missing heritability in a yeast cross. Nature, 2013 494(7436): p. 234–237. 10.1038/nature11867 23376951 PMC4001867

[9] Crow J.F., On epistasis: why it is unimportant in polygenic directional selection. Philosophical Transactions of the Royal Society B: Biological Sciences, 2010 365(1544): p. 1241–1244. 10.1098/rstb.2009.0275 20308099 PMC2871814

[10] Hansen T.F., Why epistasis is important for selection and adaptation. Evolution, 2013 67(12): p. 3501–3511. 10.1111/evo.12214 24299403

[11] Falconer D.S., Mackay T.F., and Frankham R., Introduction to Quantitative Genetics (4th edn). Trends in Genetics, 1996 12(7): p. 280.

[12] Hill W.G., Goddard M.E., and Visscher P.M., Data and theory point to mainly additive genetic variance for complex traits. PLoS Genetics, 2008 4(2): p. e1000008 10.1371/journal.pgen.1000008 18454194 PMC2265475

[13] Mäki-Tanila A. and Hill W.G., Influence of gene interaction on complex trait variation with multilocus models. Genetics, 2014 198(1): p. 355–367. 10.1534/genetics.114.165282 24990992 PMC4174947

[14] Carter A.J.R., Hermisson J., and Hansen T.F., The role of epistatic gene interactions in the response to selection and the evolution of evolvability. Theoretical Population Biology, 2005 68(3): p. 179–196. 16122771 10.1016/j.tpb.2005.05.002

[15] Cheverud J.M. and Routman E.J., Epistasis and its contribution to genetic variance components. Genetics, 1995 139(3): p. 1455–1461. 7768453 10.1093/genetics/139.3.1455 PMC1206471

[16] Wade, M.J. and C.J. Goodnight, *Perspective*: *the theories of Fisher and Wright in the context of metapopulations*: *when nature does many small experiments*. Evolution, 1998: p. 1537–1553. 10.1111/j.1558-5646.1998.tb02235.x 28565332

[17] Goodnight, C.J., *On the effect of founder events on epistatic genetic variance*. Evolution, 1987: p. 80–91. 10.1111/j.1558-5646.1987.tb05772.x 28563758

[18] Álvarez-Castro J.M. and Carlborg Ö., A unified model for functional and statistical epistasis and its application in quantitative trait loci analysis. Genetics, 2007 176(2): p. 1151–1167. 17409082 10.1534/genetics.106.067348 PMC1894581

[19] Luo L., Mao Y., and Xu S., Correcting the bias in estimation of genetic variances contributed by individual QTL. Genetica, 2003 119(2): p. 107–114. 14620950 10.1023/a:1026028928003

[20] Wu C., et al, Mimulus is an emerging model system for the integration of ecological and genomic studies. Heredity, 2007 100(2): p. 220–230. 17551519 10.1038/sj.hdy.6801018

[21] Kelly J.K., Epistasis in Monkeyflowers. Genetics, 2005 171(4): p. 1917–1931. 15944350 10.1534/genetics.105.041525 PMC1456113

[22] Kelly J., Testing the rare-alleles model of quantitative variation by artificial selection. Genetica, 2008 132(2): p. 187–198. 17607507 10.1007/s10709-007-9163-4 PMC2682333

[23] Willis J.H., The Role of Genes of Large Effect on Inbreeding Depression in Mimulus guttatus. Evolution, 1999 53(6): p. 1678–1691. 28565461 10.1111/j.1558-5646.1999.tb04553.x

[24] Bates, D., et al., *lme4*: *Linear mixed effects models using Eigen and S4**(R package v* *1**0–6)*, 2014, See http://CRAN.R-project.org/package=lme4.

[25] Van der Veen J., Tests of non-allelic interaction and linkage for quantitative characters in generations derived from two diploid pure lines. Genetica, 1959 30(1): p. 201–232. 13841036 10.1007/BF01535675

[26] Weir B. and Cockerham C.C., Two-locus theory in quantitative genetics 1977: North Carolina State University. Institute of Statistics.

[27] Kelly, J.K., *Response to selection in partially self-fertilizing populations* *I* *Selection on a single trait*. Evolution, 1999: p. 336–349. 10.1111/j.1558-5646.1999.tb03770.x 28565403

[28] Kelly J.K. and Williamson S., Predicting response to selection on a quantitative trait: a comparison between models for mixed-mating populations. Journal of theoretical biology, 2000 207(1): p. 37–56. 11027478 10.1006/jtbi.2000.2154

[29] Carlborg O. and Haley C.S., Epistasis: too often neglected in complex trait studies? Nat Rev Genet, 2004 5(8): p. 618–625. 15266344 10.1038/nrg1407

[30] Phillips P.C., Epistasis [mdash] the essential role of gene interactions in the structure and evolution of genetic systems. Nat Rev Genet, 2008 9(11): p. 855–867. 10.1038/nrg2452 18852697 PMC2689140

[31] Kempthorne O., The correlation between relatives in a random mating population. Proceedings of the Royal Society of London. Series B-Biological Sciences, 1954 143(910): p. 103–113. 13224653

[32] Cockerham C.C., An extension of the concept of partitioning hereditary variance for analysis of covariances among relatives when epistasis is present. Genetics, 1954 39(6): p. 859 17247525 10.1093/genetics/39.6.859 PMC1209694

[33] McGregor A.P., et al, Morphological evolution through multiple cis-regulatory mutations at a single gene. Nature, 2007 448(7153): p. 587–590. 17632547 10.1038/nature05988

[34] Hansen, T.F., *The evolution of genetic architecture*. Annual Review of Ecology, Evolution, and Systematics, 2006: p. 123–157.

[35] Le Rouzic, A., *Estimating directional epistasis*. Frontiers in genetics, 2014. 5. 10.3389/fgene.2014.00198 PMC4094929 25071828

[36] Phillips, P.C., S.P. Otto, and M.C. Whitlock, *Beyond the average*. Epistasis and the evolutionary process, 2000: p. 20–38.

[37] Griffing B., Theoretical consequences of truncation selection based on the individual phenotype. Australian Journal of Biological Sciences, 1960 13(3): p. 307–343.

[38] Kimura M. and Crow J.F., Effect of overall phenotypic selection on genetic change at individual loci. Proceedings of the National Academy of Sciences, 1978 75(12): p. 6168–6171. 282633 10.1073/pnas.75.12.6168 PMC393140

[39] Johansson A.M., et al, Genome-Wide Effects of Long-Term Divergent Selection. PLoS Genet, 2010 6(11): p. e1001188 10.1371/journal.pgen.1001188 21079680 PMC2973821

[40] Turner T.L., et al, Population-Based Resequencing of Experimentally Evolved Populations Reveals the Genetic Basis of Body Size Variation in Drosophila melanogaster. PLoS Genet, 2011 7(3): p. e1001336 10.1371/journal.pgen.1001336 21437274 PMC3060078

[41] Kelly J.K., Koseva B., and Mojica J.P., The genomic signal of partial sweeps in Mimulus guttatus. Genome biology and evolution, 2013 5(8): p. 1457–1469. 10.1093/gbe/evt100 23828880 PMC3762192

[42] Burke M.K., et al, Genome-wide analysis of a long-term evolution experiment with Drosophila. Nature, 2010 467(7315): p. 587–590. 10.1038/nature09352 20844486

[43] Remolina S.C., et al, GENOMIC BASIS OF AGING AND LIFE‐HISTORY EVOLUTION IN DROSOPHILA MELANOGASTER. Evolution, 2012 66(11): p. 3390–3403. 10.1111/j.1558-5646.2012.01710.x 23106705 PMC4539122

[44] Hayes B., et al, A genome map of divergent artificial selection between Bos taurus dairy cattle and Bos taurus beef cattle. Animal genetics, 2009 40(2): p. 176–184. 10.1111/j.1365-2052.2008.01815.x 19067671

[45] Simões P., et al, How repeatable is adaptive evolution? The role of geographical origin and founder effects in laboratory adaptation. Evolution, 2008 62(8): p. 1817–1829. 10.1111/j.1558-5646.2008.00423.x 18489721

[46] Orozco‐terwengel P., et al, Adaptation of Drosophila to a novel laboratory environment reveals temporally heterogeneous trajectories of selected alleles. Molecular ecology, 2012 21(20): p. 4931–4941. 10.1111/j.1365-294X.2012.05673.x 22726122 PMC3533796

[47] Scarcelli N. and Kover P.X., Standing genetic variation in FRIGIDA mediates experimental evolution of flowering time in Arabidopsis. Molecular ecology, 2009 18(9): p. 2039–2049. 10.1111/j.1365-294X.2009.04145.x 19317844

[48] Carlborg Ö., et al, Epistasis and the release of genetic variation during long-term selection. Nature genetics, 2006 38(4): p. 418–420. 16532011 10.1038/ng1761

[49] Makowsky R., et al, Beyond missing heritability: prediction of complex traits. PLoS genetics, 2011 7(4): p. e1002051 10.1371/journal.pgen.1002051 21552331 PMC3084207

