# Supplementary material for: Epistasis Is a Major Determinant of the Additive Genetic Variance in Mimulus guttatus
Source: PLoS Genet. 2015 May 6;11(5):e1005201. doi: 10.1371/journal.pgen.1005201 (PMC4422649; doi:10.1371/journal.pgen.1005201)
Supplement: S1 Table — (DOCX) [file pgen.1005201.s003.docx]

| QTL name | Linkage Group | Diagnostic Marker |
| --- | --- | --- |
| x10a | 10 | MgSTS 70, MgSTS 82 |
| X9 | 9 | MgSTS 523 |
| X1 | 1 | MgSTS 757, MgSTS 198 |
| X5a | 5 | MgSTS 40 |
| X5b | 5 | MgSTS 641/764, MgSTS 385 |
| X10b | 10 | MgSTS 308 |
| X8 | 8 | MgSTS 432, MgSTS 736,  MgSTS 31 |
