# Supplementary material for: Epistasis Is a Major Determinant of the Additive Genetic Variance in Mimulus guttatus
Source: PLoS Genet. 2015 May 6;11(5):e1005201. doi: 10.1371/journal.pgen.1005201 (PMC4422649; doi:10.1371/journal.pgen.1005201)
Supplement: S2 Table — * = 0.1 > p > 0.01; ** = 0.01 > p >0.001; *** = p < 0.001. (DOCX) [file pgen.1005201.s004.docx]

| QTL | Parameter | Corolla Width (**CW**) | Pistil Length (**Pist**) | Days to Flower (**DTF**) | Stigma-Anther Separation (**SA**) |
| --- | --- | --- | --- | --- | --- |
|  |  |  |  |  |  |
| x10a | a | 0.009 | -0.152** | 0.101 | 0.018 |
|  | d | 0.654*** | 0.17* | -0.004 | -0.051 |
| x9 | a | 0.404*** | 0.285*** | 0.166 | 0.268*** |
|  | d | 0.108 | 0.388*** | -0.111 | -0.034 |
| x1 | a | -0.076 | -0.099* | 0.144 | 0.09*** |
|  | d | 0.109 | -0.166* | -0.051 | -0.083 |
| x5a | a | 0.191* | -0.064 | -0.598** | 0.017 |
|  | d | -0.04 | -0.102 | -0.022 | -0.025 |
| x5b | a | -0.575*** | -0.037 | 0.227 | -0.023 |
|  | d | 0.092 | 0.034 | 0.367 | -0.076 |
| x10b | a | 0.362*** | 0.187*** | 0.371* | -0.043 |
|  | d | 0.794*** | -0.106 | 0.111 | 0.03 |
| x8 | a | -0.119 | -0.227*** | 0.24 | 0.019 |
|  | d | 0.313* | 0.189* | 0.181 | 0.056 |

Supplemental Table 2. * = 0.1 > p > 0.01 ; ** = 0.01 > p >0.001 ; *** = p < 0.001
