# Supplementary material for: Epistasis Is a Major Determinant of the Additive Genetic Variance in Mimulus guttatus
Source: PLoS Genet. 2015 May 6;11(5):e1005201. doi: 10.1371/journal.pgen.1005201 (PMC4422649; doi:10.1371/journal.pgen.1005201)
Supplement: S3 Table — * = 0.1 > p > 0.01; ** = 0.01 > p >0.001; *** = p < 0.001. (DOCX) [file pgen.1005201.s005.docx]

| QTL Pair | Parameter | **CW** | **Pistil** | **DTF** | **SA** |
| --- | --- | --- | --- | --- | --- |
| x10a & x1 | aa | -0.035 | 0.099* | 0.172 | -0.015 |
|  | ad | 0.171 | 0.177* | -0.49 | -0.013 |
|  | da | -0.162 | 0.009 | -0.52 | -0.033 |
|  | dd | -0.027 | -0.048 | 1.083 | 0.085 |
| x10a & x5a | aa | -0.02 | 0.005 | -0.039 | 0.005 |
|  | ad | 0.436** | 0.236* | -0.296 | 0.094* |
|  | da | -0.058 | 0.02 | -0.256 | 0.119* |
|  | dd | -0.529 | -0.259 | -0.585 | 0.047 |
| x10a & x5b | aa | 0.181* | 0.136* | -0.285 | 0.081** |
|  | ad | 0.246 | 0.092 | 0.03 | 0.052 |
|  | da | -0.027 | -0.038 | -0.343 | 0.041 |
|  | dd | -0.071 | 0.186 | -0.21 | 0.266* |
| x10a & x8 | aa | -0.04 | -0.017 | -0.184 | -0.043* |
|  | ad | 0.012 | 0.113 | -0.825** | -0.014 |
|  | da | 0.068 | 0.19* | -0.887** | 0.076 |
|  | dd | -0.49 | -0.361* | 0.563 | -0.012 |
| x9 & x1 | aa | 0.095 | 0.102* | 0.063 | 0.106*** |
|  | ad | -0.217 | 0.015 | -0.316 | 0.118* |
|  | da | 0.269 | 0.035 | -0.7* | -0.048 |
|  | dd | 0.194 | 0.026 | 0.268 | 0.15 |
| x9 & x5a | aa | -0.089 | -0.09* | 0.066 | -0.085** |
|  | ad | -0.048 | 0.107 | -0.309 | -0.032 |
|  | da | 0.297 | 0.11 | -0.046 | 0.094 |
|  | dd | 0.112 | -0.194 | 0.69 | 0.045 |
| x9 & x5b | aa | -0.14* | -0.034 | -0.096 | -0.068** |
|  | ad | 0.121 | 0.263** | -0.365 | 0.064 |
|  | da | -0.045 | -0.128 | 0.141 | -0.109* |
|  | dd | 0.201 | -0.237 | 0.247 | -0.06 |
| x9 & x8 | aa | -0.015 | 0.149** | -0.403* | -0.002 |
|  | ad | -0.254 | -0.156 | 0.214 | -0.1* |
|  | da | 0.161 | -0.091 | 0.481 | 0.044 |
|  | dd | 0.207 | -0.055 | -0.903 | 0.273* |
| x1 & x5b | aa | 0.084 | 0.026 | 0.223 | -0.027 |
|  | ad | -0.05 | 0.066 | -0.631* | -0.038 |
|  | da | -0.179 | 0.024 | 0.213 | -0.008 |
|  | dd | -0.337 | 0.024 | -0.558 | 0.135 |
| x1 & x10b | aa | -0.045 | -0.027 | -0.283* | -0.045* |
|  | ad | -0.179 | -0.192* | 0.436 | -0.029 |
|  | da | -0.118 | -0.147 | -0.071 | 0.078 |
|  | dd | -0.279 | -0.196 | -0.415 | -0.117 |
| x10b & x8 | aa | -0.158* | -0.14** | -0.242 | 0.048* |
|  | ad | 0.23 | 0.092 | 0.01 | 0.004 |
|  | da | -0.256 | 0.031 | -0.064 | -0.083 |
|  | dd | -0.681* | -0.116 | 0.204 | -0.058 |

Supplemental Table 3. * = 0.1 > p > 0.01 ; ** = 0.01 > p >0.001 ; *** = p < 0.001
