# Supplementary material for: Epistasis Is a Major Determinant of the Additive Genetic Variance in Mimulus guttatus
Source: PLoS Genet. 2015 May 6;11(5):e1005201. doi: 10.1371/journal.pgen.1005201 (PMC4422649; doi:10.1371/journal.pgen.1005201)
Supplement: S4 Table — (DOCX) [file pgen.1005201.s006.docx]

| EFFECTS |  |  |  |  |  |  |
| --- | --- | --- | --- | --- | --- | --- |
| Effect 1 | Effect 2 | Correlation | Count | Lower 95% | Upper 95% | P-value |
| Pist | CW | 0.465 | 58 | 0.2349 | 0.6458 | 0.0002 |
| DTF | CW | -0.0039 | 58 | -0.2619 | 0.2546 | 0.9768 |
| DTF | Pist | -0.2844 | 58 | -0.5055 | -0.0281 | 0.0305 |
| SA | CW | 0.2082 | 58 | -0.0529 | 0.4427 | 0.1167 |
| SA | Pist | 0.2865 | 58 | 0.0305 | 0.5073 | 0.0292 |
| SA | DTF | -0.1353 | 58 | -0.3803 | 0.1274 | 0.3111 |

Supplemental Table 4. Correlations between traits for effect estimates.
