# Supplementary material for: Epistasis Is a Major Determinant of the Additive Genetic Variance in Mimulus guttatus
Source: PLoS Genet. 2015 May 6;11(5):e1005201. doi: 10.1371/journal.pgen.1005201 (PMC4422649; doi:10.1371/journal.pgen.1005201)
Supplement: S5 Table — (DOCX) [file pgen.1005201.s007.docx]

| TRAITS |  |  |  |  |  |  |
| --- | --- | --- | --- | --- | --- | --- |
| Trait 1 | Trait 2 | Correlation | Count | Lower 95% | Upper 95% | P-value |
| CWmm | day1 | 0.2997 | 10430 | 0.2821 | 0.3171 | <.0001 |
| SA_mm | day1 | -0.0689 | 10027 | -0.0884 | -0.0494 | <.0001 |
| SA_mm | CWmm | 0.1646 | 10045 | 0.1455 | 0.1836 | <.0001 |
| pist_mm | day1 | 0.2433 | 10125 | 0.2249 | 0.2615 | <.0001 |
| pist_mm | CWmm | 0.4656 | 10144 | 0.4502 | 0.4807 | <.0001 |
| pist_mm | SA_mm | 0.3202 | 10037 | 0.3026 | 0.3377 | <.0001 |

Supplemental Table 5. Correlations between phenotypic traits.
