# Supplementary material for: Epistasis Is a Major Determinant of the Additive Genetic Variance in Mimulus guttatus
Source: PLoS Genet. 2015 May 6;11(5):e1005201. doi: 10.1371/journal.pgen.1005201 (PMC4422649; doi:10.1371/journal.pgen.1005201)
Supplement: S1 Fig — (DOCX) [file pgen.1005201.s008.docx]

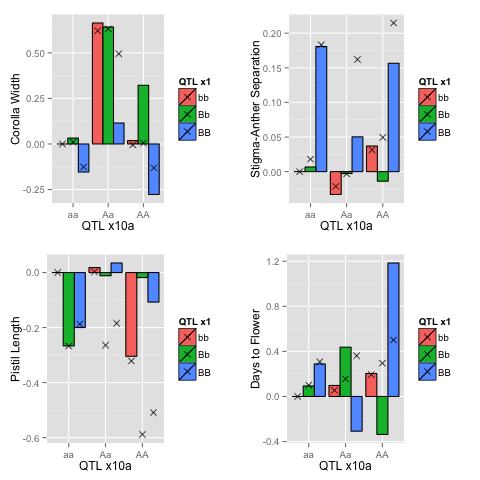


Supplemental Figure 1.


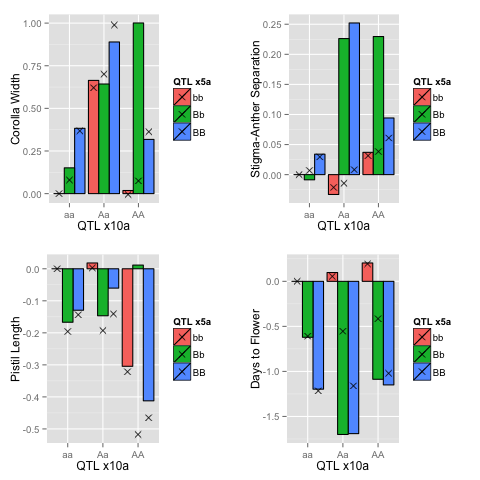


Supplemental Figure 1.


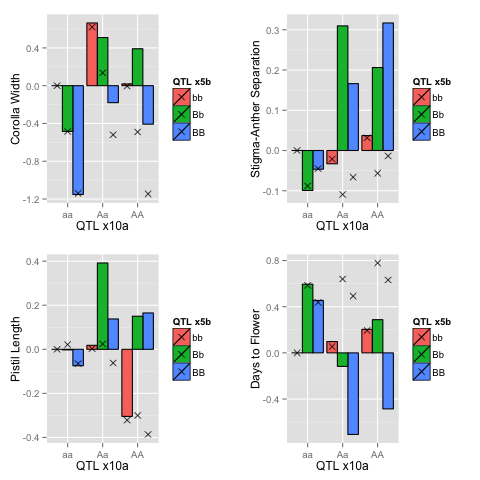


Supplemental Figure 1.


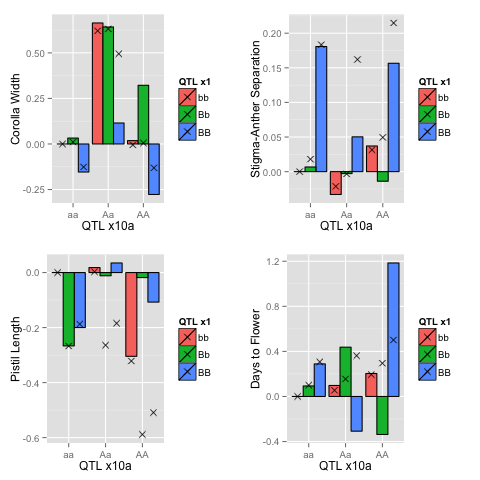


Supplemental Figure 1.


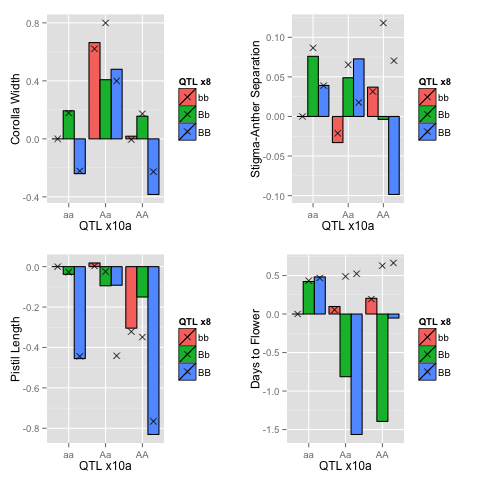


Supplemental Figure 1.


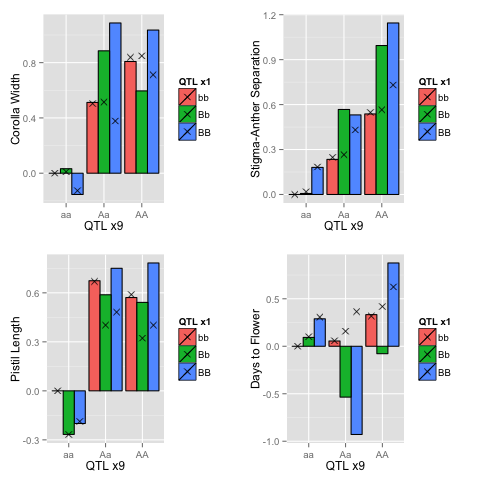


Supplemental Figure 1.


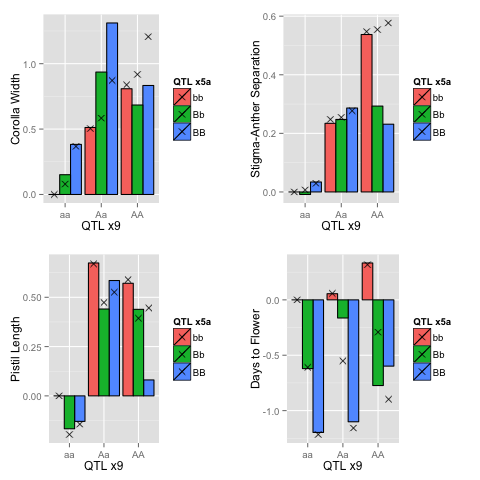


Supplemental Figure 1.


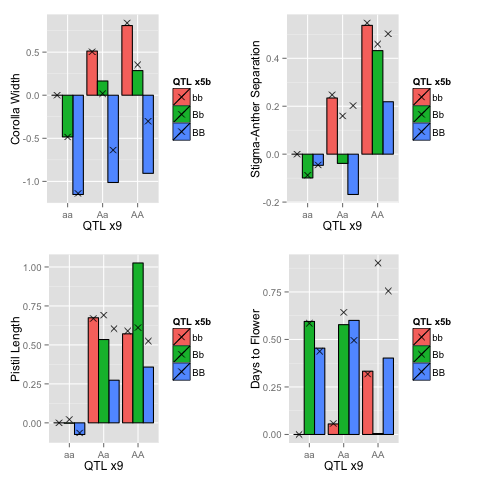


Supplemental Figure 1.


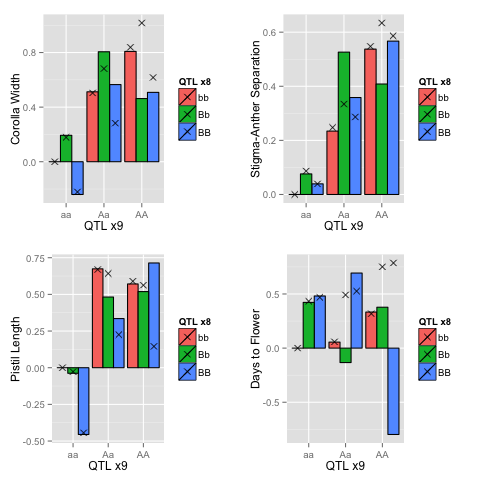


Supplemental Figure 1.


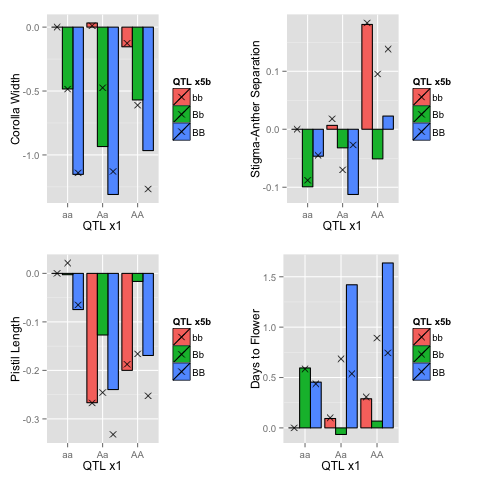


Supplemental Figure 1.


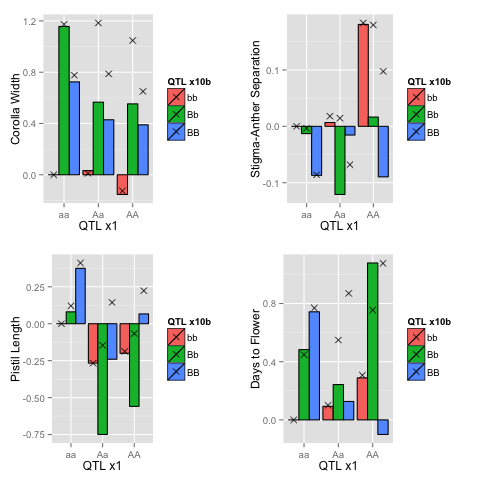


Supplemental Figure 1.


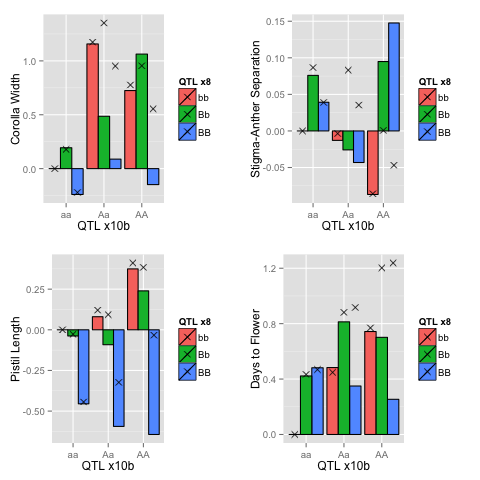


Supplemental Figure 1.
