# Supplementary figures and images for: Epistasis Is a Major Determinant of the Additive Genetic Variance in Mimulus guttatus
Source: PLoS Genet. 2015 May 6;11(5):e1005201. doi: 10.1371/journal.pgen.1005201 (PMC4422649; doi:10.1371/journal.pgen.1005201)

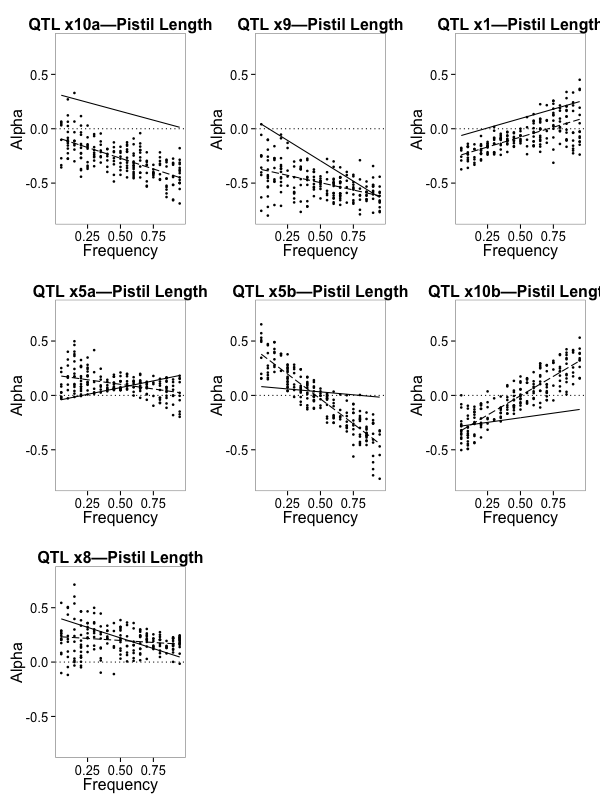


Supplemental Figure 2.


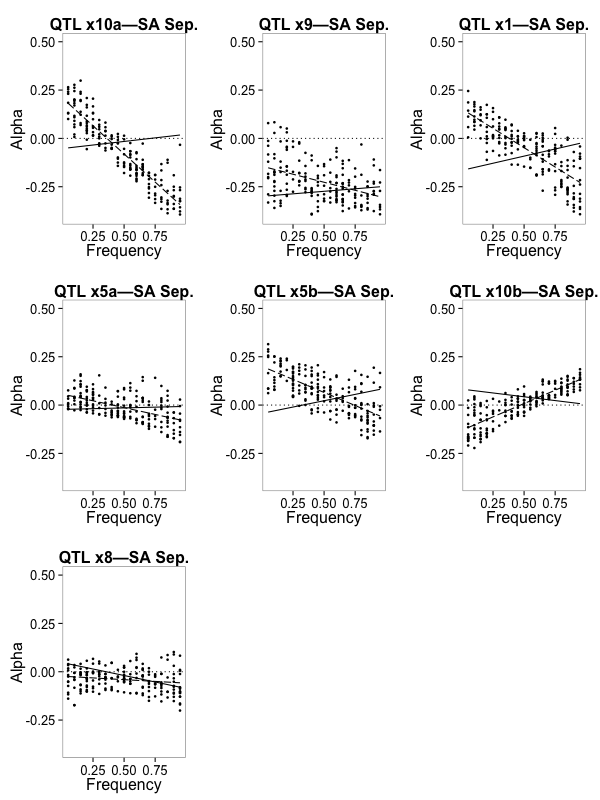


Supplemental Figure 2.


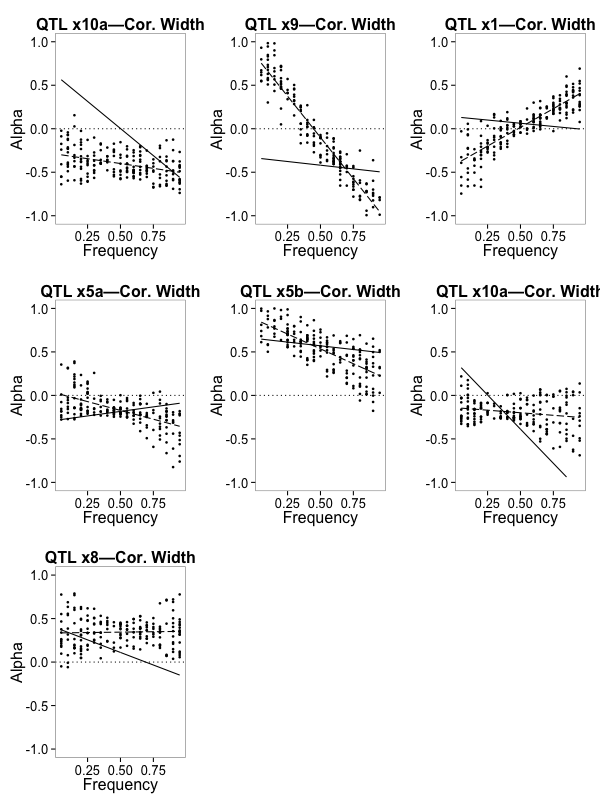


Supplemental Figure 2.


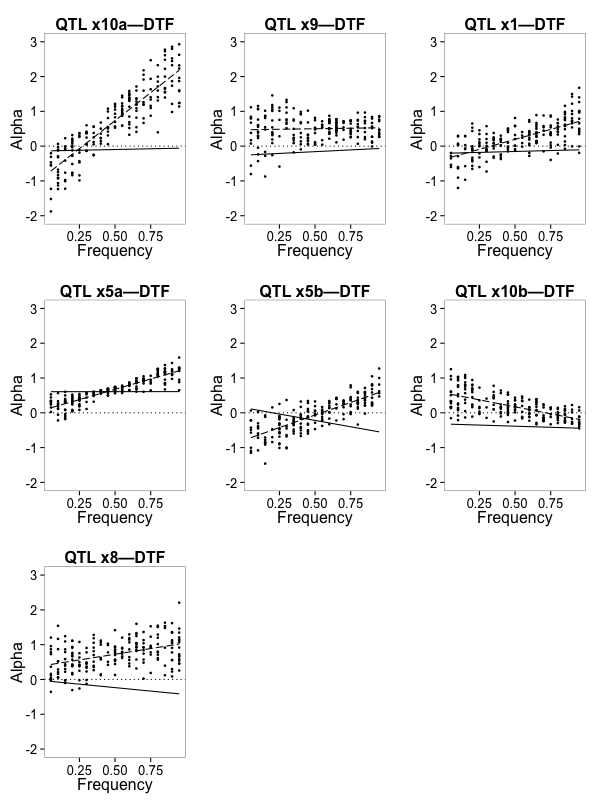


Supplemental Figure 2.

Supplement: S2 Fig — The black line indicates the alpha value calculated without epistasis whereas the blue line shows the best-fit line through the scatter of points, which are the alpha values calculated with epistasis included. (DOCX) [file pgen.1005201.s009.docx]

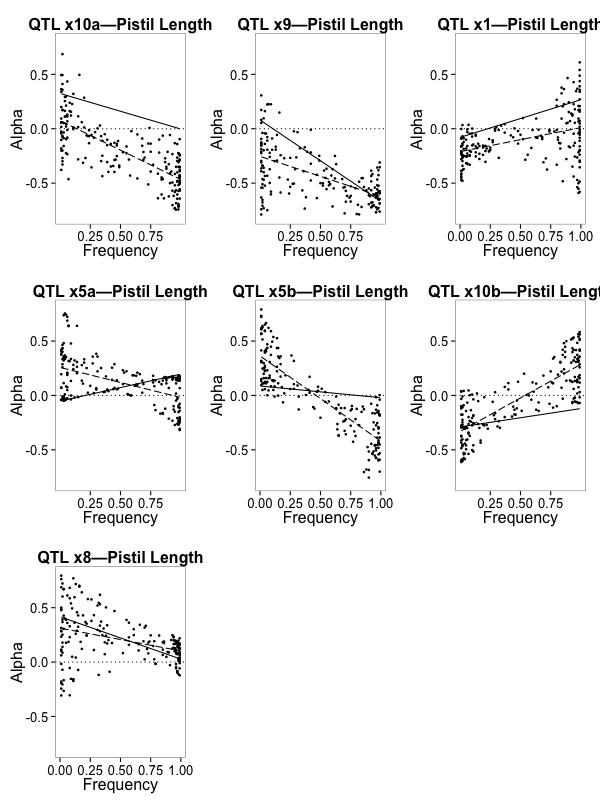


Supplemental Figure 3.


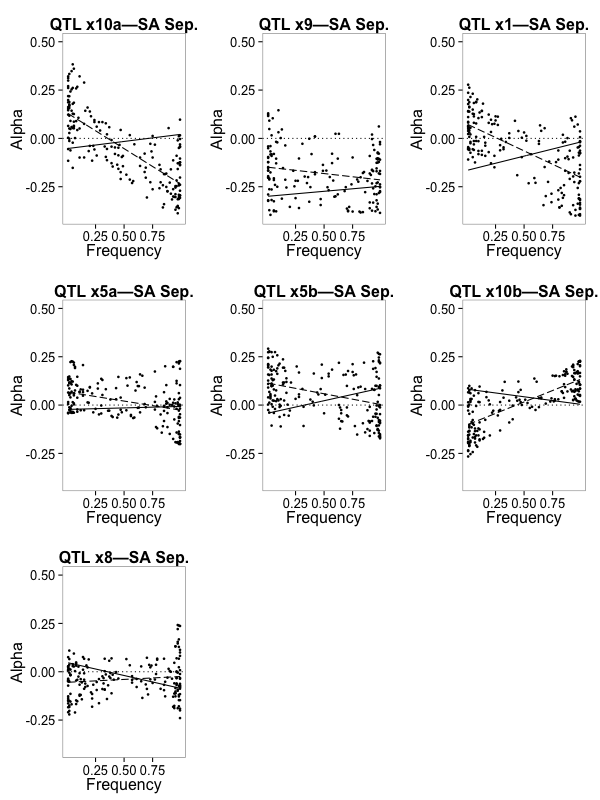


Supplemental Figure 3.


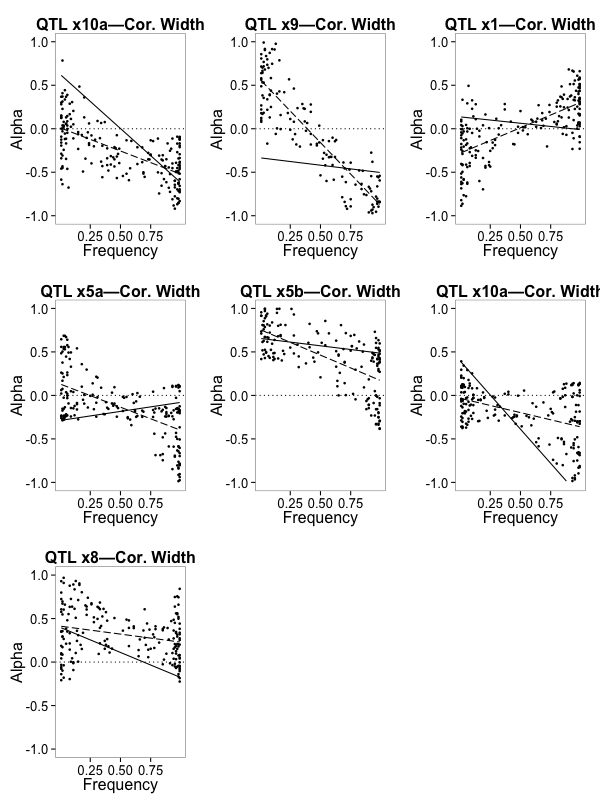


Supplemental Figure 3.


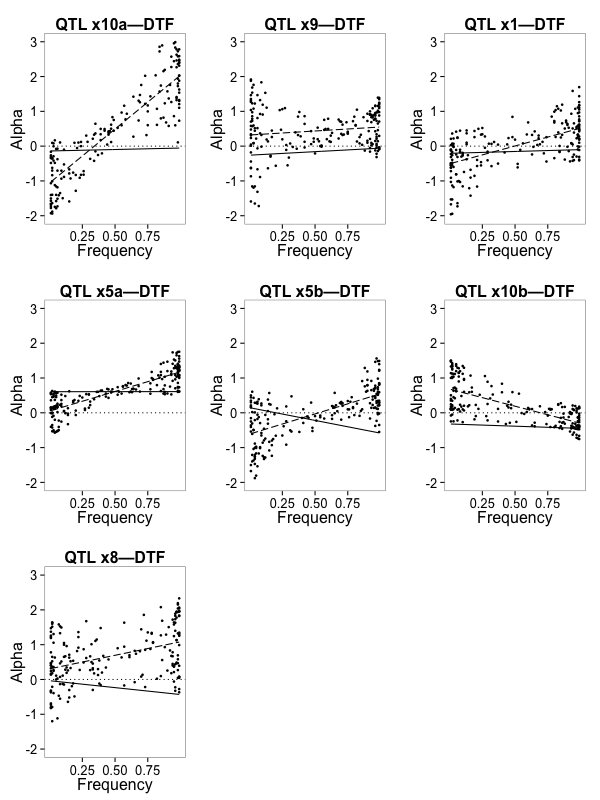


Supplemental Figure 3.

Supplement: S3 Fig — The black line indicates the alpha value calculated without epistasis whereas the blue line shows the best-fit line through the scatter of points, which are the alpha values calculated with epistasis included. (DOCX) [file pgen.1005201.s010.docx]
