# Supplementary material for: Epistasis Is a Major Determinant of the Additive Genetic Variance in Mimulus guttatus
Source: PLoS Genet. 2015 May 6;11(5):e1005201. doi: 10.1371/journal.pgen.1005201 (PMC4422649; doi:10.1371/journal.pgen.1005201)
Supplement: S10 Fig — (DOCX) [file pgen.1005201.s017.docx]

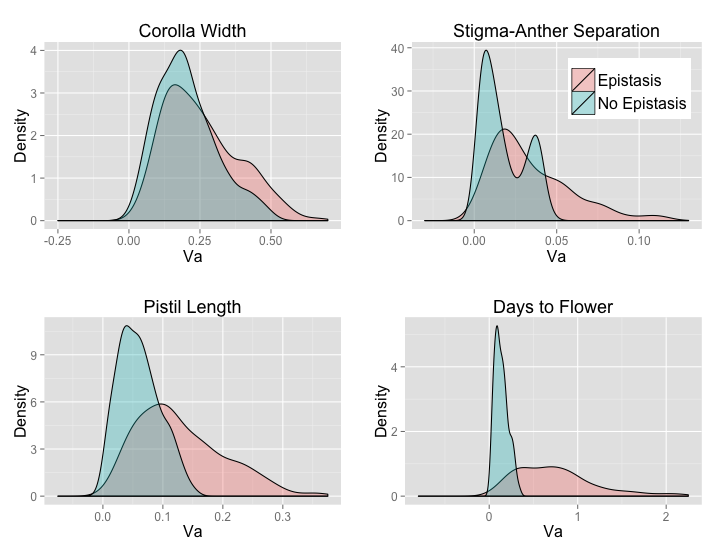


Supplemental Figure 10. Distributions for uncorrected additive genetic variance for the U-shaped distribution of allele frequencies.
