# Supplementary material for: Epistasis Is a Major Determinant of the Additive Genetic Variance in Mimulus guttatus
Source: PLoS Genet. 2015 May 6;11(5):e1005201. doi: 10.1371/journal.pgen.1005201 (PMC4422649; doi:10.1371/journal.pgen.1005201)
Supplement: S11 Fig — The black arrows indicate the ‘true’ value predicted using the effects from which data was simulated. (DOCX) [file pgen.1005201.s018.docx]

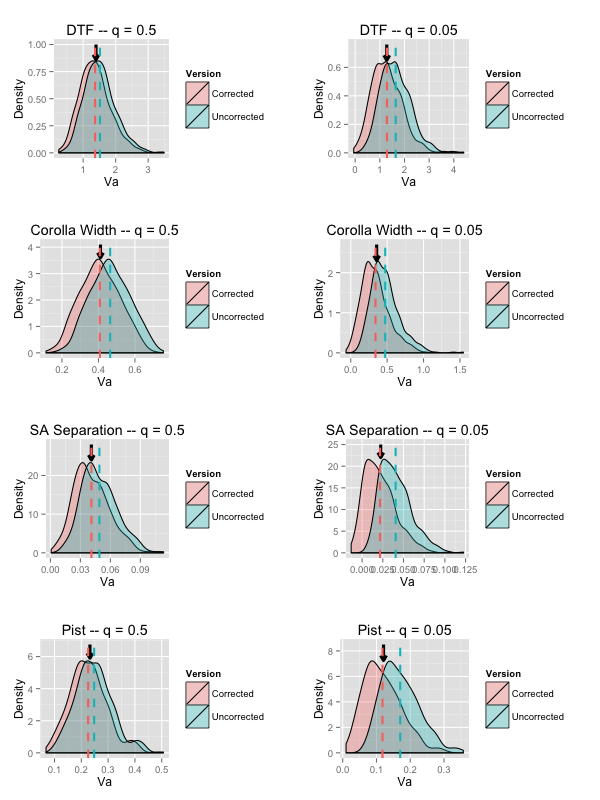


Supplemental Figure 11. Distributions for additive genetic variances calculated with and without bias-correction for the bias-correction simulations. The black arrows indicate the ‘true’ value predicted using the effects from which data was simulated.
